# Transcriptomic and proteomic approach to identify differentially expressed genes and proteins in *Arabidopsis thaliana* mutants lacking chloroplastic 1 and cytosolic FBPases reveals several levels of metabolic regulation

**DOI:** 10.1186/s12870-016-0945-7

**Published:** 2016-12-01

**Authors:** Mauricio Soto-Suárez, Antonio J. Serrato, José A. Rojas-González, Rocío Bautista, Mariam Sahrawy

**Affiliations:** 1 Departamento de Bioquímica, Biología Molecular y Celular de Plantas, Estación Experimental del Zaidín, Consejo Superior de Investigaciones Científicas, C/Profesor Albareda 1, 18008 Granada, Spain; 2 Plataforma Andaluza de Bioinformática/SCBI, Edificio de Bioinnovación, Parque Tecnológico de Andalucía, Universidad de Málaga, C/ Severo Ochoa 34, 29590 Campanillas, Spain; 3 Present address: Corporación Colombiana de Investigación Agropecuaria, CORPOICA, Km 14 vía Mosquera, Mosquera, Cundinamarca Colombia

**Keywords:** Fructose-1,6-bisphosphatase, Carbohydrate, Transcriptomic, Proteomic, Rosette, Root

## Abstract

**Background:**

During the photosynthesis, two isoforms of the fructose-1,6-bisphosphatase (FBPase), the chloroplastidial (cFBP1) and the cytosolic (cyFBP), catalyse the first irreversible step during the conversion of triose phosphates (TP) to starch or sucrose, respectively. Deficiency in cyFBP and cFBP1 isoforms provokes an imbalance of the starch/sucrose ratio, causing a dramatic effect on plant development when the plastidial enzyme is lacking.

**Results:**

We study the correlation between the transcriptome and proteome profile in rosettes and roots when *cFBP1* or *cyFBP* genes are disrupted in *Arabidopsis thaliana* knock-out mutants. By using a 70-mer oligonucleotide microarray representing the genome of Arabidopsis we were able to identify 1067 and 1243 genes whose expressions are altered in the rosettes and roots of the *cfbp1* mutant respectively; whilst in rosettes and roots of *cyfbp* mutant 1068 and 1079 genes are being up- or down-regulated respectively. Quantitative real-time PCR validated 100% of a set of 14 selected genes differentially expressed according to our microarray analysis. Two-dimensional (2-D) gel electrophoresis-based proteomic analysis revealed quantitative differences in 36 and 26 proteins regulated in rosettes and roots of *cfbp1*, respectively, whereas the 18 and 48 others were regulated in rosettes and roots of *cyfbp* mutant, respectively. The genes differentially expressed and the proteins more or less abundant revealed changes in protein metabolism, RNA regulation, cell signalling and organization, carbon metabolism, redox regulation, and transport together with biotic and abiotic stress. Notably, a significant set (25%) of the proteins identified were also found to be regulated at a transcriptional level.

**Conclusion:**

This transcriptomic and proteomic analysis is the first comprehensive and comparative study of the gene/protein re-adjustment that occurs in photosynthetic and non-photosynthetic organs of Arabidopsis mutants lacking FBPase isoforms.

**Electronic supplementary material:**

The online version of this article (doi:10.1186/s12870-016-0945-7) contains supplementary material, which is available to authorized users.

## Background

In leaves, assimilated carbon is either transiently stored as starch in the chloroplasts or exported to sink tissues in the form of sucrose, synthesized in the cytosol. To maintain an optimum photosynthetic rate, this carbon partitioning needs to be highly regulated [1]. This regulation is strongly dependent on the circadian rhythm of the plant and carbon metabolite levels and is carried out through the export of triose phosphate intermediates produced in the chloroplast during the reductive pentose phosphate pathway or the Calvin-Benson cycle [2]. In C_3_ plants, several key enzymes are necessary for a highly coordinated carbon metabolism. These include fructose-1,6-bisphosphatase (FBPase), of which three isoforms have been reported [3]. A cytosolic enzyme (cyFBP), present both in prokaryotic and eukaryotic cells, participates in gluconeogenesis and sucrose synthesis [4]. A chloroplastic isoform (cFBP1), also found in photosynthetic eukaryotes [5], is regulated by the reduction of disulphide bonds via thioredoxin (TRX) as well as by changes in the pH and Mg^2+^ concentration that results from illumination [3]. Recently, another plastidial cFBP isoform (cFBP2) was identified in our laboratory [6]. In contrast to cFBP1, this novel isoform lacks the regulatory redox domain required for activation by TRX. A fraction of the triose phosphates is used to produce ribulose-1,5-bisphosphate for Calvin-Benson cycle regeneration via a cFBP1 and the remainder can be exported to the cytosol to be converted to sucrose via cyFBP. The function of cFBP2 in sucrose synthesis and the control of carbohydrate distribution still has to be elucidated.

It has been reported that the reduction in cFBP1 activity in Arabidopsis (*Arabidopsis thaliana*) plants leads to an increase in soluble sugar [7]. More recently, Rojas-Gonzalez and coworkers [8] have characterized physiologically and metabolically the loss-of-function mutants *cyfbp* and *cfbp1.* The knock-out *cfbp1* line shows a dwarf phenotype, chlorotic leaves, a low photosynthesis rate, and a high sucrose/starch rate. On the other hand, *cyfbp* displays a wild-type phenotype, a decreased photosynthesis capacity, and high starch synthesis and mobilization rates [8]. Finally, simultaneous over-expression of a triose phosphate/phosphate translocator and *cyFBP* in Arabidopsis causes increases in soluble sugars and starch contents [9]. Other works highlight the key role of FBPases in the control of the sucrose/starch balance [3].

In Arabidopsis and plants of agricultural interest, the balance between the distribution and utilization of carbohydrates (sucrose and starch) has been studied using wild-type plants [10] and knock-out mutants of transcriptional or redox regulators of primary-metabolism enzymes [11–14], starchless mutants [15–17], and combining the generation of knock-out mutants and whole-genome microarray analyses [18–22]. Thousands of genes showed significant transcript changes in Arabidopsis starchless mutants lacking the chloroplastic isoform of phosphoglucomutase (PGM) gene when compared with wild-type plants at different time-points during diurnal light/dark cycles [19, 22, 23]. Nevertheless, little is known about the proteome profiling in plant carbohydrate metabolism. Considering the importance that FBPases have in plant-carbohydrate homeostasis, in this study we performed a genome-wide mRNA- and protein-profiling analysis comparing rosette and root organs from Arabidopsis *cfbp1* and *cyfbp* knock-out mutants with wild-type plants. We found that: (i) *cfbp1* and *cyfbp* mutants affect the expression of a broad range of genes, representing the reprogramming of near to 10% of the Arabidopsis genome; (ii) *cFBP1* or *cyFBP* gene disruption induces different expression profiles in rosettes and roots; (iii) differentially expressed genes/proteins are related to carbon metabolism, protein metabolism, cell signalling, gene regulation, transport, and stress responses; and (iv) the transcriptome and the proteome data were correlated.

## Results

### Differentially expressed genes in Arabidopsis knock-out mutants lacking *cFBP1 *and *cyFBP *genes

To analyse the genome-wide effects of *cFBP1* or *cyFBP* gene disruption in rosettes and roots tissues, we performed a microarray analysis comparing *cfbp1* and *cyfbp* knock-out mutants with the wild-type plants using a 70-mer oligonucleotide microarray representing the genome of Arabidopsis. A bootstrap analysis with Significance Analysis of Microarrays (SAM) was used to identify differentially expressed genes. SAM calculates the fold change and significance of differences in expression. The delta values ranged from 1.07 to 1.86 for each comparison. The false significant number (FSN) ranged between 13.7 and 23.7, while the false discovery rate (FDR) ranged from 1.10 to 2.20. Of the 28,964 protein-coding gene transcripts analysed, 4457 genes were found to be differentially expressed, of which 3,198 and 1,259 corresponded to up- and down-regulated genes, respectively (Table 1). There was a total of 2,310 and 2,147 differentially regulated genes in both *cfbp1* and *cyfbp* mutants, respectively, representing the reprogramming of 8.0 and 7.4% of the total evaluated transcriptome (Table 1). The *cfbp1* mutation was associated with 474 and 1,057 up-regulated genes in rosettes and roots, respectively, whereas 726 and 941 genes showed an increased expression pattern in rosettes and roots of the *cyfbp* background. For down-regulated expression profiles, 593 and 186 down-regulated genes were identified in rosettes and roots of the *cfbp1* background, respectively, whereas 342 and 138 genes were down-regulated in rosettes and roots of the *cyfbp* background (Table 1). Notably, the roots showed very few down-regulated genes (15 and 13%, respectively) in comparison to the up-regulated genes (85 and 87%, respectively) reported in both FBPase mutants. A full list of significantly altered transcripts (cut-off of 1.5-fold changes) is presented in Additional file 1: Table S1.

**Table 1:** Statistical summary of significance analyses of microarrays

Gene expression	*cfbp1* mutant		*cyfbp* mutant	
	Rosettes	Roots	Rosettes	Roots
Delta value	1.61	1.23	1.07	1.86
False significant number (FSN)	16.00	13.7	18.1	23.7
False discovery rate (FDR)	1.5	1.1	1.7	2.2
Up-regulated	474 (44%)	1057 (85%)	726 (68%)	941 (87%)
Down-regulated	593 (56%)	186 (15%)	342 (32%)	138 (13%)
Total	1067	1243	1068	1079

The number of up- and down-regulated genes that are differentially expressed in rosettes and roots tissues in both *cfbp1* and cy*fbp* mutants when compared with wild-type plants

**Fig. 1:**
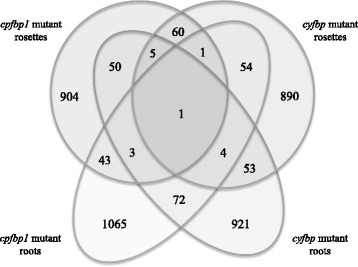
Differential gene expression in *cfbp1* and *cyfbp* mutants. Venn diagram showing the overlap of genes differentially expressed in *cfbp1* and *cyfbp* mutants. Most of regulated genes are mutant and tissue specific

Considering the fact that *cFBP1* and *cyFBP* are both key genes in the carbohydrate metabolism, one can postulate that a high proportion of the differentially expressed genes identified in our microarray experiments should be sharing between *cfbp1* and *cyfbp*. To determine whether *cFBP1* or *cyFBP* gene disruption induces similar gene-expression-profile changes, we compared the differentially expressed genes between mutants and plant organs (Fig. 1). As shown in the Venn diagram, most of the genes are specifically regulated in rosettes and roots of the respective genetic backgrounds. Of the 4,457 *fbp*-regulated genes, 904 and 1,065 genes are regulated only in rosettes and roots of *cfbp1*, respectively, whereas 890 and 921 others are exclusively regulated in rosettes and roots of *cyfbp*, respectively and might be considered as genes which are responding differentially in rosettes and roots in both *cfbp1* and *cyfbp* mutants. However, those genes which are specifically regulated in *cfbp1* and *cyfbp* belong to the same functional categories, as discussed below, the most abundant groups corresponded to (i) genes with no assigned biological process; (ii) protein synthesis, turnover, and destination; and (iii) RNA regulation, processing, and binding. It means that although differentially expressed genes are specific to each mutant, the same functional biological processes are affected.

### Cluster analysis of microarray data

**Fig. 2:**
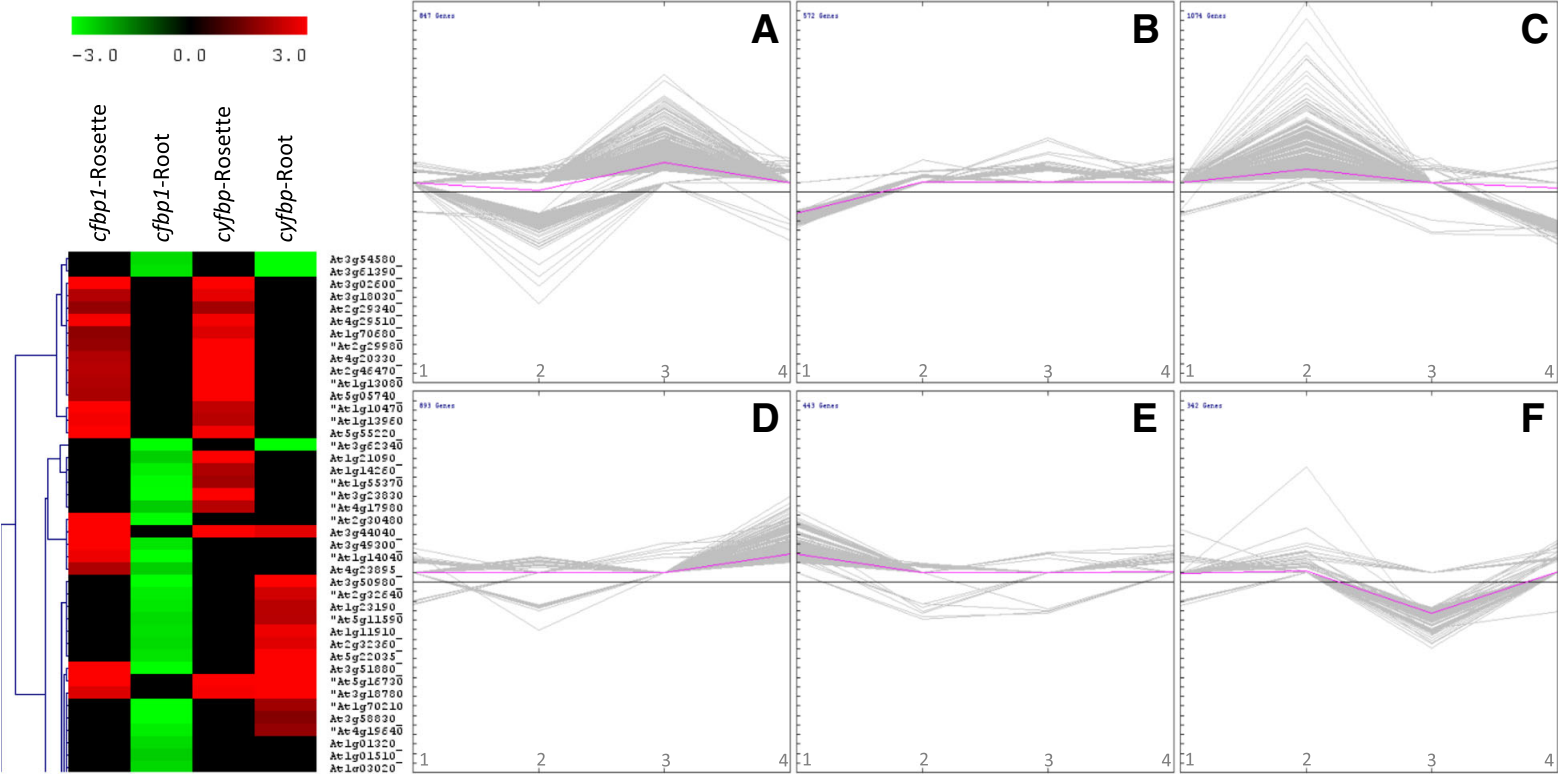
Clusters of transcripts based on patterns of differential expression. A representative fragment of clustering analysis showing the behaviour of each gene relative to the others in rosettes and roots of *cfbp1* and *cyfbp* mutants (*left*). The figure shows up-regulated genes in rosettes of *cfbp1* and *cyfbp*, down-regulated genes in *cfbp1* roots; and up- or down-regulated genes in *cyfbp* roots. Differentially expressed transcripts were clustered, using the *k*-means method. Six Clusters were created (Clusters **a**-**f**, *right side*), with Clusters **a** and **d** comprising up-regulated genes in rosettes and roots of *cyfbp*, respectively; and Clusters **f** and **c** comprising down-regulated genes. Clusters **e** and **b** comprising up- and down-regulated genes in *cfbp1* rosettes. Clusters **c** and **a** comprising up- and down-regulated genes in *cfbp1* roots, respectively. The x-axis represents (*1*) *cfbp1* rosettes (*2*) *cfbp1* roots (*3*) *cyfbp* rosettes, and (*4*) *cyfbp* roots. The y-axis represents the expression level

A *k*-means clustering analysis was performed to gain an overview of the performance of each differentially expressed gene, compared with the others in the rosettes and roots of both the *cfbp1* and *cyfbp* mutants. Six clusters were defined (Fig. 2, genes are identified in Additional file 2: Table S2).

Cluster A includes 847 transcripts with *cyfbp* rosette up-regulated expression and down-regulated gene expression in *cfbp1* roots (Fig. 2a and Additional file 2: Table S2A). Of these transcripts, 19.3% encode hypothetical or unknown proteins. The cluster includes known genes which are involved in the Calvin cycle, glycolysis, or starch metabolism, such as the coding for glyceraldehyde 3-phosphate dehydrogenase (At1g79530), *cFBP1* gene (At3g54050), beta-amylase 5 and 6 (At2g32290 and At4g15210), and ADP-glucose pyrophosphorylase large subunit 4 (At2g21590). One striking finding was that transcripts encoding proteins required for sugar transport (e.g. sugar transporter 2 – STP2, UDP-Galactose transporter 6 and STP4) were enriched only in Clusters A, B and C, which are constituted mainly by up-regulating genes in *cyfbp* rosettes and *cfbp1* roots. Cluster B contained 572 transcripts, 19.0% of which encoded hypothetical or unknown proteins (Fig. 2b and Additional file 2: Table S2B). These transcripts had mainly down-regulated expression in *cfbp1* rosettes. Cluster-B transcripts include genes involved in glycolysis, gluconeogenesis, and starch metabolism such as pyrophosphate-dependent 6-phosphofructose-1-kinase (At1g12000), pyruvate kinase (At5g08570), and beta-amylase 4 (*BAM4*; At5g55700). Cluster C contains 1,074 transcripts, most of them with up-regulated expression in *cfbp1* roots (Fig. 2c and Additional file 2: Table S2C). These transcripts exhibited peak expression in *cfbp1* roots with down-regulated expression in *cyfbp* roots. Approximately 17.0% of the Cluster-C transcripts encode hypothetical or unknown proteins. Transcripts that encode proteins which are essential in the Calvin cycle are present in this cluster. For example, fructose-bisphosphate aldolase (At4g38970), glyceraldehyde-3-phosphate dehydrogenase B (At1g42970) and the ribulose bisphosphate carboxylase small chain (At1g67090). Cluster D includes 897 transcripts with high expression in *cyfbp* roots. Of these transcripts, 13.0% encode hypothetical or unknown proteins. It is worth mentioning that a large number of up-regulated genes encoding for enzymes involved in glycolysis and gluconeogenesis fell into this cluster (Additional file 2: Table S2). These include phosphoglycerate kinase (At3g12780), phosphoglucomutase (At1g23190), glucose-6-phosphate isomerase (At5g42740), PFK7 and PFK2-phosphofructokinase (At5g56630 and At5g47810), glyceraldehyde-3-phosphate dehydrogenase (At3g04120), 2,3-bisphosphoglycerate mutase 1 (At1g09780), and pyruvate kinase (At5g63680).

Clustering analysis also revealed groups of co-ordinately expressed genes in rosettes of *cfbp1* and *cyfbp*. Genes that were up-regulated in *cfbp1* rosettes and down-regulated in *cyfbp* were represented by clusters E and F, respectively (Fig. 2e and f and Additional file 2: Table S2). The percentage of the transcripts encoding hypothetical proteins or unknown proteins was 19.1% in Cluster E and 22.2% in Cluster F. Curiously, there were no genes associated with sucrose or starch metabolism in cluster F.

### Biological processes affected by FBPase genes disruption

**Fig. 3:**
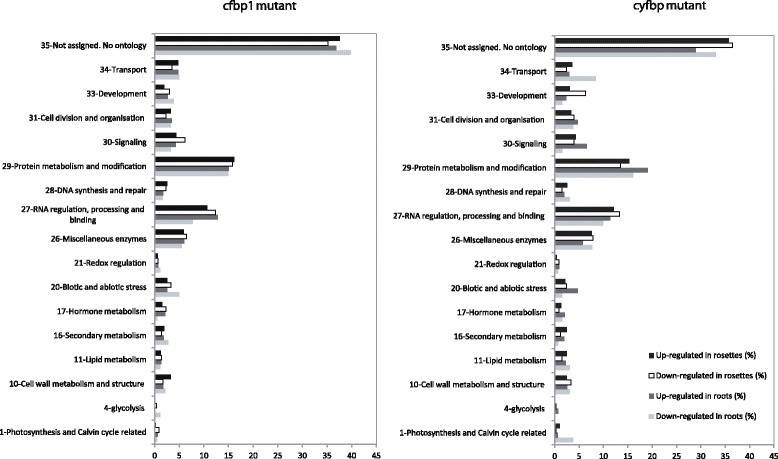
MapMan bin membership for differentially expressed genes in *cfbp1* and *cyfbp*. Differentially expressed genes with their differential expression values were imported into MapMan software version 3.5.0. Seventeen functional category bins were created. The bin numbers and their corresponding bin name are graphed on the y-axis. Percentage of probe sets in each bin is graphed on the x-axis

Next, we investigated which metabolic and cellular processes were affected by *cFBP1* or *cyFBP* inactivation. For this, the final list of regulated genes with their differential expression values was imported into MapMan 3.5.0 [18, 21] together with the mapping file containing TAIR Arabidopsis whole genome annotation. Since the correspondence between GO terms and MapMan bins is not trivial, we have finally discarded these approaches. But we performed the statistical analysis of functional categories (bins) with the Over-Representation Analysis of PageMan (Additional file 3: Figure S1). This is a classical statistical test for classes: given the number of objects chosen, the total number of objects, and the class size, the test provides a statistical evidence (based on contingency tables) to discern which object (e.g., gene) from a class (e.g., functional bins) is not classified by chance. Therefore, the numbers of genes included in bins are those that the ORA analyses considered over-represented in the differentially expressed sets. Thus, differentially expressed genes were assigned to 17 functional category bins (Fig. 3). Most differentially expressed genes (more than 60%) in both *cfbp1* and *cyfbp* fell into three classes: (i) genes with no assigned biological process; (ii) protein synthesis, turnover, and destination; and (iii) RNA regulation, processing, and binding. Miscellaneous enzymes were well represented (6-8%). Cell signalling, cell organization, and carbon metabolism and transport, together with biotic and abiotic stress, represented more than 20% of the differentially regulated genes (Fig. 3).

#### Photosynthesis, photorespiration, and carbon metabolism related genes

**Fig. 4:**
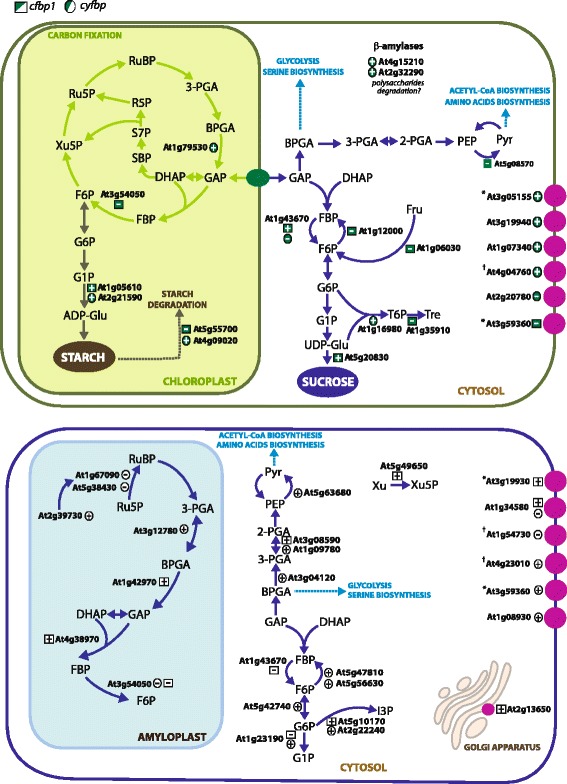
Schematic representation of regulated genes involved in carbohydrate metabolism. A model of starch and sucrose metabolism is represented. The model shows genes (AGI numbers) revealing an altered expression profile in *cfbp1* rosettes and roots; and *cyfbp* rosettes and roots. Rosette regulated genes (*upper panel*) are numbered as follows: glyceraldehyde 3-phosphate dehydrogenase (At1g79530), *cFBP1* (At3g54050), ADP-glucose pyrophosphorylase large subunit 4 (At2g21590), beta-amylase 4 (At5g55700), alpha-isoamylase 3 (At4g09020), four sugar transporter family proteins B amylase 5 and 6 (At2g32290 and At4g15210), pyruvate kinase (At5g08570), pyrophosphate-fructose-6-phosphate 1-phosphotransferase (At1g12000), pfkB-like carbohydrate kinase (At1g06030), trehalose-phosphatase synthase 2 (At1g16980), trehalose-6-phosphate phosphatase (At1g35910). Root regulated genes (lower panel) are: ribulose bisphosphate carboxylase small chain (At1g67090), rubisco activase (At2g39730), Rubisco small subunit (At5g38430), phosphoglycerate kinase (At3g12780), Glyceraldehyde-3-phosphate dehydrogenase B (At1g42970), fructose-bisphosphate aldolase (At4g38970), *cFBP1* (At3g54050), carbohydrate transmembrane transporter (At1g08930), pyruvate kinase (At5g63680), 2,3-bisphosphoglycerate mutase (At3g08590), glyceraldehyde-3-phosphate dehydrogenase (At3g04120), xilulose kinase 2 (At5g49650), *cyFBP* (At1g43670), phosphofructokinases PFK2 (At5g47810) and PFK7 (At5g56630); glucose-6-phosphate isomerase (At5g42740), inositol-3-phosphate synthase (At5g10170), inositol-3-phosphate synthase (At2g22240). (+) indicates up-regulated, and (-) indicates down-regulated. Pink circles indicate genes which are supposed to be located in plastid or plasma membranes, according to Cell eFP Browser prediction for subcellular localization (http://bar.utoronto.ca/cell_efp/cgi-bin/cell_efp.cgi). (*) predicted as plasma membrane and/or Golgi apparatus transporters; (†) three putative localizations: plasma membrane, Golgi apparatus, and plastid

The differentially expressed genes involved in photosynthesis, photorespiration, and the Calvin cycle identified in this study were up- and down-regulated in rosettes and roots of *cfbp1*, respectively (Fig. 4; Additional file 4: Table S3). In leaf the genes ADP-glucose pyrophosphorylase small subunit 2 (APS2; At1g05610) and sucrose synthase (At5g20830) are up- regulated and the genes chlorophyll A-B binding protein (At1g76570), photosystem II reaction centre PsbP protein (PsbP, At4g15510), and photosystem I subunit IV protein (At4g28750) are down-regulated (Additional file 1: Table S1). Root up-regulated genes encoding for fructose-bisphosphate aldolase (At4g38970), glyceraldehyde-3-phosphate dehydrogenase B (At1g42970) and 2,3-bisphosphoglycerate mutase (At3g08590) (Additional file 1: Table S1). Curiously, though in a non-photosynthetic organ, root up- and down-regulated loci included genes encoding for photosystem I reaction centre subunit psaK protein (At1g30380), phytochromobilin:ferredoxin oxidoreductase (At3g09150) and photosystem II reaction centre W protein (At2g30570). In the case of *cyfbp*, most of the differentially expressed genes were up-regulated in rosette leaves, as the coding for glyceraldehyde 3-phosphate dehydrogenase (At1g79530), photosystem I reaction centre subunit III protein (At1g31330) and phytochromobilin:ferredoxin oxidoreductase (At3g09150), and up- or down-regulated in roots such as ferredoxin-NADP^+^ reductase (At4g32360), rubisco activase (At2g39730) and phosphoglycerate kinase 1 (At3g12780), and the ribulose bisphosphate carboxylase small chain (At1g67090), respectively. Notably, *cyFBP* (At1g43670) was found to be up-regulated in *cfbp1* rosettes (Fig. 4). This result was previously reported by Rojas-Gonzalez and co-workers [8].

It is also worth noting that a large number of key genes encoding for enzymes associated with glycolysis and gluconeogenesis showed altered expression profiles in *cfbp1* and *cyfbp* genetic backgrounds (Fig. 4; Additional file 1: Table S1). It appears that transcript accumulation for an enzyme catalysing the opposite reaction of FBPase, pyrophosphate-fructose-6-phosphate 1-phosphotransferase (At1g12000), was down-regulated in *cfbp1* rosette leaves together with pyruvate kinase (At5g08570). Nevertheless, specific root transcriptional re-adjustment included the up- and down-regulation of 2,3-bisphosphoglycerate mutase (At3g08590) and phosphoglucomutase (At1g23190), respectively. The *cyfbp* root tissue showed a rise in transcript levels of genes encoding for phosphoglucomutase (At1g23190), glucose-6-phosphate isomerase (At5g42740), PFK7 and PFK2-phosphofructokinase (At5g56630 and At5g47810), the cytosolic glyceraldehyde-3-phosphate dehydrogenase (At3g04120), 2,3-bisphosphoglycerate mutase 1 (At1g09780), and pyruvate kinase (At5g63680), while an increase of chloroplastic glyceraldehyde-3-phosphate dehydrogenase (At1g79530) was detected in *cyfbp* rosettes (Fig. 4). Surprisingly, whatever the inactivated FBPase isoform, the above-mentioned genes all encoded for enzymes participating in cytosolic biochemical pathways.

Some genes involved in sucrose biosynthesis, such as sucrose synthase (At5g20830), and *cyFBP* (At1g43670*)*, were up-regulated in rosettes of *cfbp1* mutant, whereas starch-degradation related genes, e.g. beta-amylase 4 (*BAM4*; At5g55700), were down-regulated in rosettes and up-regulated in roots. In contrast, genes encoding for enzymes involved in starch metabolism were up-regulated both in rosettes as well as in roots of *cyfbp*. Mutant studies show that the gene alpha-isoamylase 3 (At4g09020) is strongly involved in starch breakdown whilst ADP-glucose pyrophosphorylase large subunit 4 (At2g21590) is related to starch synthesis [24] (Fig. 4). Several of the proteins encoded by the root differentially-expressed genes were found among the 289 proteins identified by Balmer *et al.* [25] in the amyloplast of wheat endosperm. Most of them are involved in carbohydrate and nitrogen metabolism, cell division, stress, signaling and transport.

#### Redox regulation and stress responses

More than 30 genes involved in the redox status were regulated in the two FBPase-lacking mutants. Most of these encode for glutaredoxins, thioredoxins or proteins associated with ascorbate and glutathione metabolism. In general, glutaredoxin-related genes (At5g58530, At3g02000, At4g28730) were negatively affected in rosettes or roots of *cfbp1*, whilst thioredoxins (At1g53300, At1g21750, At3g16110) and ascorbate-glutathione-related genes were up-regulated (Additional file 1: Table S1). Most of the redox-associated genes were up-regulated in *cyfbp* rosettes and roots (Additional file 1: Table S1). These genes include thioredoxin reductase B (At4g35460), thioredoxin ATY1 (At1g76760) cytochrome reductase (At5g20080), thioredoxin-like 1-3 (At2g33270), and catalase 3 (At1g20620). Furthermore, 135 genes associated with biotic and abiotic stress have also been identified in *cfbp1* and *cyfbp* backgrounds. The proportions of up- or down-regulated genes were relatively similar in *cfbp1* and *cyfbp* rosettes and in *cfbp1* roots, while *cyfbp* roots had only two down-regulated (At1g42560 and At5g66910) and 44 up-regulated genes (Additional file 1: Table S1 and Additional file 5: Table S4).

Classification based on MapMan, and Gene Ontology and corroborated with PageMan has shown that *cFBP1* or *cyFBP* inactivation affected the whole-genome expression levels in a wide range of molecular functions and biochemical pathways. However, it was helpful to find that the functional category biotic and abiotic stress was well represented (Additional file 6: Figure S2). Of the loci responding to stress responses, 31 and 35 genes were identified as regulated in rosettes and roots of the *cfbp1* background, respectively, whereas 23 and 46 genes were regulated in rosettes and roots of the *cyfbp* background (Additional file 1: Table S1; Additional file 5: Table S4). To understand the significance of this result more clearly, we performed a gene-clustering analysis comparing all differentially expressed genes found in *cfbp1* and *cyfbp* with five stress-related experiments that used the Arabidopsis oligonucleotide microarrays. These data sets are available in GEO data repository and correspond to transcriptional analyses of Arabidopsis subjected to abiotic stress by arsenate [26], Cu^2+^ (accession number GSE13114), drought, and combined drought and heat stress [27], and subjected to biotic stress by *Tobacco etch potyvirus* (TEV) infection [28]. Comparisons of these data sets reveal that *cfbp1-* and *cyfbp*-regulated genes are also differentially expressed in response to biotic and abiotic stress (Figures S2 and S3). We also conducted k-means clustering analysis to group the regulated genes from all experiments according to the similarity of their expression patterns, using MeV software with the default options. Four and six clusters were defined after comparison of rosette and root *fbp*-regulated genes with the other data sets, respectively (Additional file 7: Figure S3).

#### RNA regulation, processing and binding

In rosettes and roots, *cFBP1* and *cyFBP* gene disruptions induce a highly dynamic transcriptional regulation. One hundred transcription factors were found as differentially expressed in *cfbp1* rosettes, 119 in *cfbp1* roots, 112 in *cyfbp* rosettes and 96 in *cyfbp* roots (Additional file 5: Table S4). This means that 9.4 and 9.6% of the differentially expressed genes found in *cfbp1* and *cyfbp*, respectively, coded for transcription factors. These transcription factors belong mainly to AP2/EREBP, bZip, bHLH, MYB, GATA, WRKY, C2C2(Zn) DOF, and C2H2 zinc finger family proteins and probably could regulate upstream components of the transcriptional response to *cFBP1* or *cyFBP* gene inactivation.

#### Cell signalling

Over 220 receptor kinases, soluble protein kinases, Ser/Thr protein phosphatases, MAP kinase pathway components, calcium binding, and G-proteins showed alteration of their respective transcript levels in *cfbp1* and *cyfbp* backgrounds. Among these, 55 genes were affected in *cfbp1* rosettes, 50 in *cfbp1* roots, 43 in *cyfbp* rosettes, and 63 in *cyfbp* roots (Additional file 5: Table S4). These proteins are known to play pivotal roles in regulating and coordinating aspects of metabolism, cell growth, cell differentiation, and cell division [29]. The implication of Ser/Thr protein phosphatases in the control of the redox reactions of photosynthesis has recently been documented [30]. In *cfbp1* rosettes, the proportion of up- or down-regulated genes was similar; nevertheless, most of the cell-signalling-related genes are up-regulated in *cfbp1* roots and *cyfbp* rosettes.

Over 75 genes assigned to hormone metabolism and signalling showed modified expression profiles in *cfbp1* and *cyfbp* (Additional file 5: Table S4). Most were auxin- or gibberellins-regulated genes, followed by abscisic acid or ethylene-response genes. Only 19 genes were down-regulated, 13 of them in *cfbp1* rosettes, whilst the rest were up-regulated.

#### Protein synthesis, turnover, and destination

Protein synthesis, degradation, and modification group is the second-best-represented category after the one assigned to genes with unknown biological functions. In this category, most of the regulated genes are involved in protein degradation, particularly those involved in the ubiquitin pathway, followed by genes associated with protein synthesis and post-translational modification. Sixty-three genes belonging to the ubiquitin pathway were differentially expressed in *cfbp1* rosettes, 57 in *cfbp1* roots, 63 in *cyfbp* rosettes, and 68 in *cyfbp* roots (Additional file 5: Table S4). Our results indicate that protein-degradation machinery plays an important role in *cfbp1* and *cyfbp* mutants; it can be part of the normal protein-turnover process but can also play a role in an ubiquitin complex involved in signalling via protein degradation.

#### Transport

The transcript levels of several genes involved in amino acid, peptide, calcium, phosphate, membrane, and particularly sugar transport were altered in *cfbp1* and *cyfbp* backgrounds (Fig. 4). A gene encoding for UDP-galactose transporter 6 (At3g59360) was down-regulated in *cfbp1* rosettes, while sugar transporter 4 (At3g19930), GDP-mannose transmembrane transporter 1 (At2g13650), and a putative monosaccharide transporter (At1g34580) were up-regulated in *cfbp1* roots (Additional file 1: Table S1 and Additional file 5: Table S4). Sugar transporter 2 (At1g07340) and three sugar transporter family proteins (At3g05155, At4g04760, At3g19940) were up-regulated in *cyfbp* rosettes, and only a mannitol transporter (At2g20780) was down-regulated. Finally, a carbohydrate transmembrane transporter (At1g08930) and two UDP-galactose transporters (At4g23010 and At3g59360) were up-regulated in *cyfbp* roots, whereas two monosaccharide transporters were down-regulated in this organ (At1g34580 and At1g54730) (Additional file 1: Table S1 and Additional file 5: Table S4).

### Validation of differentially expressed genes, using QRT-PCR

To validate the *cfbp1* and *cyfbp* microarray results, we performed Quantitative Real-Time PCR (QRT-PCR) on a set of 14 genes, representing different functional categories, which were up- or down-regulated in rosette and root (Table 2). The genes selected for QRT-PCR belonged to the most representatives functional categories, such as protein synthesis, degradation, and post-translational modification (At2g20140), RNA regulation, processing and binding (At3g61850), glycolysis and gluconeogenesis (At4g15210, At5g20830 and At1g50460), photosynthesis and Calvin-cycle-related genes (At1g79530 and At2g39730), transport (At1g07340 and At3g19930), development (At5g24780), redox regulation (At1g76760 and At1g28480), miscellaneous enzymes (At5g20340), and unassigned biological process (At1g67850). The QRT-PCR results supported microarray data, and also showed that the gene-expression pattern was identical for all genes tested. As shown in Table 2, the expression values were higher when QRT-PCR was used in relation to the data provided by the microarray, indicating that real time PCR is a more sensitive method.

**Table 2:** Validation by QRT-PCR of differentially expressed genes

			Array				QRT-CR			
			*cpfbp1*		*cyfbp*		*cpfbp1*		*cyfbp*	
Gene	Putative function	Primer sequence	Rosette	Root	Rosette	Root	Rosette	Root	Rosette	Root
At2g39730	Rubisco activase	5'ACGTCCAGCTCCCAGGAA 3'ACTCTCGCCCTCAAAGCA	**1.2** ±0.08	**0.0** ±0.02	**0.0** ±0.09	**2.4** ±0.07	**1.6** ±0.1	**0.0** ±0.1	**-0.5** ±0.2	**2.9** ±0.1
At3g19930	sugar transporter 4	5' TATGTGGCGGCTTTGGTGTCTT 3' TTGGGCAAAGCCGTTGAAAGCA	**0.0** ±0.04	**1.9** ±0.1	**1.4** ±0.04	**0.0** ±0.1	**-0.2** ±0.1	**22.0** ±0.3	**6.4** ±0.2	**0.0** ±0.1
At1g67850	unknown protein	5'CTCCCTCCATGTGCTGCA 3'TTCCCGTCTGTCGCCTCA	**0.0** +-0.1	**1.6** ±0.01	**0.6** +-0.01	**0.0** +-0.1	**0.2** +-0.1	**2.7** ±0.2	**-0.2** +-0.1	**0.0** ±0.1
At2g20140	26S protease regulatory complex subunit 4	5'TGAGCCAGGCACTGGGAA 3'CGCTTGGTGCCAACAGCA	**0.7** ±0.1	**0.0** ±0.02	**0.0** ±0.03	**2.2** ±0.08	**1.2** ±0.1	**-0.2** ±0.1	**-0.8** ±0.2	**2.7** ±0.2
At3g61850	DAG1 (DOF affecting germination 1)	5' ACCAACAACAACACACCGCA 3' TTTCTCTTGTGGCCTCGCCTTT	**2.8** ±0.01	**0.0** ±0.01	**1.2** ±0.1	**2.6** ±0.01	**3.1** ±0.2	**0.0** ±0.1	**2.3** ±0.2	**2.6** ±0.2
At1g50460	HKL1 hexokinase	5'ACAGTTGTTGCGGTAGAAGGAGGT 3'AGAGCAGAGCCAATGCTAGAACCA	**0.0** ±0.01	**0.0** ±0.01	**0.0** ±0.01	**3.7** ±0.02	**0.0** ±0.1	**-0.9** ±0.1	**0.0** ±0.1	**31.6** ±0.3
At5g20340	beta-1,3-glucanase 5	5' TTTGATGCGTTCGTGTGGGCAA 3' AACCCGTCGATGCCTTTGTT	**0.0** ±0.01	**0.0** ±0.02	**0.0** ±0.01	**3.5** ±0.05	**0.0** ±0.1	**0.0** ±0.1	**0.0** ±0.1	**3.9** ±0.3
At1g28480	GRX480 glutaredoxin protein	5'ACGACAACCGTCGGGGAA 3'CGCCGCCTTGAACTCCAA	**2.5** ±0.01	**0.0** ±0.01	**0.0** ±0.01	**0.0** ±0.01	**3.1** ±0.2	-	**0.5** ±0.3	-
At1g76760	Thioredoxin AtY1	5' TGGTCCTTGCCAGTTCATGGTT 3' AGCACCCTCAAAGCGATCACAA	**0.0** ±0.02	**0.0** ±0.02	**4.0** ±0.1	**0.0** ±0.01	**0.0** ±0.1	-	**4.9** ±0.2	-
At5g20830	sucrose synthase 1	5' ACAGCCAACGTGAGCGTTTGAA 3' TGGCTTCAACCCTGGAAAGCAA	**2.3** ±0.1	**0.0** ±0.02	**0.0** ±0.07	**0.0** ±0.02	**3.0** ±0.2	-	**0.5** ±0.1	-
At5g24780	VSP1 (vegetative storage protein 1)	5'TACGGTCTCCCACGTCCA 3'AAGGTGCCAGCTTCTGCA	**-2.3** ±0.05	**0.0** ±0.02	**0.0** ±0.02	**0.0** ±0.06	**-11.2** ±0.1	-	**0.0** ±0.1	-
At1g07340	ATSTP2 (sugar transporter 2)	5' ATGGTGTGAACGCAATCGCT 3' AATACAATCAGCGGCACGGCAT	**0.0** ±0.02	**0.0** ±0.01	**2.8** ±0.05	**0.0** ±0.05	**0.5** ±0.1	-	**2.9** ±0.2	-
At1g79530	GAPCP-1; glyceraldehyde-3-phosphate dehydrogenase	5' TGCAAGAAGTGTGCAACCCA 3' ATGTTGCAATGCGGAGGACCAA	**0.0** ±0.05	**0.0** ±0.03	**2.4** ±0.3	**0.0** ±0.01	**0.3** ±0.1	-	**2.6** ±0.1	-
At4g15210	beta-amylase	5' AATGTGGTGGAAACGTTGGCGA 3' AACAGCGGTTCTTCCGGCAAAT	**0.0** ±0.01	**0.0** ±0.02	**2.3** ±0.01	**0.0** ±0.06	**0.3** ±0.1	-	**2.4** ±0.1	-

List of genes used to validate the *cfbp1* and *cyfbp* expression changes as determined by microarray analysis. Averages of fold-change (in bold) expression for each gene (normalized using the 18S ribosomal gene) are indicated. (-) indicates not tested

### Differentially regulated proteins from rosettes and roots of *cfbp1 *and *cyfbp *mutants

**Fig. 5:**
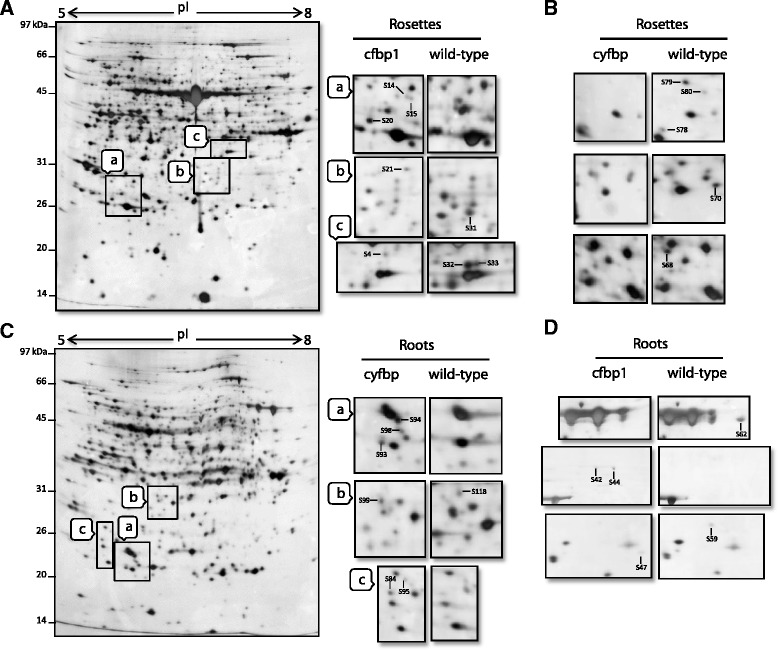
2-DE images from rosette and root tissues of *cfbp1* and *cyfbp* mutants. 2-DE gels of total proteins from rosette and root tissues of *cfbp1* and *cyfbp* mutants. The indicated portions of the gel, **a** through **c**, are reproduced in enlarged windows, **a** through **c**, of 2-DE gels for each mutant and tissue. (**a**) 2-DE gel of total proteins from rosette tissue of *cfbp1* mutant and enlarged panels, *cfbp1* (*left*) and WT plants (*right*). (**b**) Portions of selected regions of 2-DE gels showing rosette tissue of *cyfbp* mutant against WT plants. (**c**) 2-DE gel of total proteins from root tissue of *cyfbp* mutant and enlarged panels, *cyfbp* (left) and WT plant (*right*). (**d**) Selected regions of 2-DE gels showing root tissue of *cfbp1* mutant against WT plants. Protein spots indicated (S4–S118) were identified by MALDI-TOF/TOF analysis (Table S4). The figure shows representative experiments carried out three times

Microarrays provide an almost totally comprehensive assessment of the transcriptome that is not necessarily reflected at the protein or functional levels, and therefore we then pursued a proteomic approach to study protein profiles in *fbp* mutants. The analysis of the 2-DE pattern revealed a total of 128 different protein-spot intensities out of about 1000 that were resolved in each image (Additional file 8: Table S5). From these spots, 36 and 26 corresponded to proteins regulated in rosettes and roots of *cfbp1*, respectively, whereas the 18 and 48 others were regulated in rosettes and roots of *cyfbp* mutant, respectively (details of statistics, Mr/pI and protein function in Additional file 8: Table S5)*.* Figure 5 shows representative experiments (at least two biological replicates) for the determination of up- and down-regulated proteins in rosettes and roots from *fbp* mutants through the analysis of the protein spots picked and identified by MS (Additional file 8: Table S5). These spots correspond to proteins with putative functions in general metabolism, photosynthesis, protein synthesis, protein destination, signalling, RNA regulation, hormone metabolism, redox regulation, cell organization, development, biotic and abiotic stress, and miscellaneous enzymes and proteins with unknown functions (Additional file 9: Figure S4).

As we expected, our proteomic analyses demonstrated that the lack of *cFBP1* and *cyFBP* also triggers changes at the protein level. In the case of the *cfbp1* mutant, among the up- or down-regulated protein found in rosettes and roots, several are involved in photosynthesis and carbohydrate metabolism: glyceraldehyde-3-phosphate dehydrogenase C2 (At1g13440) and rubisco activase (At2g39730) were up-regulated in rosettes, whereas the granule-bound starch synthase (At1g32900) was down-regulated in roots. Furthermore, a number of up- or down regulated proteins (Additional file 8: Table S5) are also involved in biotic and abiotic stress, such as: glutathione s-transferase (At4g19880), a well-known marker of stress involved in reactive oxygen species (ROS) detoxifying processes [31]; AtNDX18 (At1g14860), a member of the nudix hydrolase family of proteins that helps protect against oxidative DNA and RNA damage in plant cells [32]; the TUDOR-SN protein 2 (At5g61780), which is essential for stress tolerance and stabilizes the levels of stress-responsive mRNAs; monodehydroascorbate reductase (At1g63940), which is associated with salt tolerance through scavenging of ROS [33]; the hypersensitive-induced response protein 3 (At3g01290); cold-shock protein 2 (At4g38680); the ATHVA22H protein (At1g19950), which is induced under stress by drought, chilling, and high salinity [34]; and zeaxanthin epoxidase (At5g67030), which is involved in chlorophyll protection against oxidative damage [35]. In relation to the protein spots in *cyfbp* background identified, some are also associated with photosynthesis and carbohydrate metabolism: soluble starch synthase (At5g24300), phosphoenolpyruvate carboxykinase (At4g37870), GDP-L-fucose synthase (At1g17890) were down-regulated in *cyfbp* rosettes. Glyceraldehyde-3-phosphate dehydrogenase B (At1g42970), phosphoenolpyruvate carboxylase (At3g14940), alanine aminotransferase (At1g72330), 6-phosphogluconate dehydrogenase (At5g41670), and beta-glucosidase (At3g09260) were up-regulated in *cyfbp* roots, whereas fructokinase (At2g31390) and ketose-bisphosphate aldolase (At1g18270) were down-regulated. Some 35% of the protein spots identified were predicted to be plastid-localized, indicating that *cFBP1* or *cyFBP* gene disruption affects mainly proteins associated with plastids. It is worth noting that the functional category “redox regulation” was better represented in proteomic data (10%) when compared with microarray data sets (<3%). It has been reported that the correlation between mRNA and protein abundance varies by gene functional group [36], implying that the translation-level regulation of redox-related proteins might be critical in *cfbp1* and *cyfbp* mutants. By contrast, the proteins related to the ubiquitin-proteasome pathway appeared well represented in both microarray and proteomic data sets, suggesting that the regulation of protein degradation via the ubiquitin-proteasome system might be important both at the transcriptome and proteome levels.

### Congruence of cfbp1 and cyfbp array data with proteome analysis

We compared the *cfbp1*- and *cyfbp*-regulated proteins identified by 2-DE in rosettes and roots with the differentially regulated transcripts detected by microarray analysis. Protein spots corresponding to 32 of the 128 identified differentially expressed spots had differential RNA accumulation (see Additional file 8: Table S5). From these protein spots, seven were regulated in *cfbp1* rosettes, 5 in *cfbp1* roots, 3 in *cyfbp* rosettes, and 17 in *cyfbp* roots. Differentially expressed genes and protein levels changes identified simultaneously by using transcriptome analysis and proteomics technology fell into important functional categories, including cell redox homeostasis (At5g61510, At1g28480, At3g52880, At2g44160, At3g25530, At1g75280, At2g44160), protein process (At1g54270, At3g13920, At2g20140, At2g45240, At5g50920), metabolic process (At2g39730, At1g42970, At5g43780, At1g17890, At1g72330, At1g54220) cell signalling (At1g10630, At2g28940, At5g63400, At1g17170, At1g16920, At5g61780), and RNA regulation (At5g24780).

## Discussion

Carbohydrate metabolism and its regulation are complex phenomena that may be driven by the coordinate expression of numerous genes. Transcription profiling and proteomics data are not available for *cfbp1* and *cyfbp* mutants and, therefore, this study provides a compilation of in-depth transcriptome and proteome effects of *cFBP1* or *cyFBP* gene disruption, and can help elucidate the importance of the different steps regulated by these enzymes in controlling carbohydrate metabolism and, indirectly, other processes in Arabidopsis. Our results show that about 10% of the total evaluated transcriptome was affected by *cFBP1* or *cyFBP* gene disruption. It has been previously reported that a perturbation in the carbohydrate balance can affect a high number of genes involved in general metabolic reactions, signalling, gene regulation, and protein metabolism. Osuna et al. [19] reported changes in transcript levels for >1,700 genes after 3 h of 15 mM sucrose addition to Arabidopsis plants. A comprehensive analysis revealed radical changes at the metabolic, proteomic, and overall transcriptional levels during a circadian cycle and extended night in wild-type Arabidopsis plants and starchless *pgm* mutant [19, 22, 23]. Bläsing et al. [20] showed that more than 17% of the genes presented more than a two-fold larger diurnal change in the starchless *pgm* mutant when compared with Col-0. The large percentage of affected genes found in our analysis, together with major phenotypic and physiological changes [8], reflects the severe impact of omitting both cFBP1 as cyFBP activities from the metabolic scene and the physiological adaptations derived from the lack of both enzymes. Similarly, Usadel et al. [18], on conducting an overall transcriptome analysis during a circadian cycle in wild-type and starchless *pgm* mutant plants, observed a decrease in transcripts for genes involved in biosynthesis and cellular growth but an increase for genes involved in the remobilization of alternative carbon sources. PageMan statistical analyses of our study have shown differentially expressed functional categories. The classification by functional categories shows that expression changes of genes related to protein metabolism and modification, RNA regulation, signalling, photosynthesis, carbon metabolism, biotic and abiotic stress, and redox regulation were more susceptible to *cFBP1* or *cyFBP* gene inactivation than were other genes.

Comparative transcriptomic analyses revealed that in addition to the metabolism-related genes, other mechanisms might contribute to explain the phenotypic differences and developmental alterations in *cFBP1* compared to *cyFBP* [8]. The *cfbp1* mutant presents a smaller rosette size, low chlorophyll content and CO_2_-assimilation rate in relation to wild-type plants that trigger in a lesser rosette development, a lower content of soluble sugars, less starch accumulation, and a greater superoxide dismutase (SOD) activity [8]. The mutant also had some developmental alterations, including stomatal opening defects and increased numbers of root vascular layers. For example, a significant number of the *cfbp1* differently regulated genes encoded proteins that were related to cell wall biosynthesis/modification, development and redox regulation (Fig. 3). It is important to note that the lack of *cFBP1* and *cyFBP* is leading to an important impact on genes related to protein metabolism, RNA regulation; and biotic and abiotic stress as showed in Fig. 3. This result is in line with the phenotype observed for *cfbp1* [8]. Furthermore, cFBP1 and cyFBP are key players in sugar metabolism in rosettes and roots from Arabidopsis. Previous studies have reported that reduced cFBP1 activity results in a lower total-sugar content but showed a higher sucrose:starch ratio in relation to control plants [7]. If the step catalysed by cFBP1 is disrupted, there is a deficit of starch synthesis that the plant would counterbalance by inducing genes related to starch synthesis or reducing those of the starch degradation. In agreement with this hypothesis, a number of genes differentially expressed in this mutant (Fig. 4 and Additional file 4: Table S3) are associated with starch metabolism, glycolysis, and the Calvin-Benson cycle. These data suggest that *cFBP1* inactivation could trigger changes of down-stream pathways related to starch synthesis such as the induction of APS2 (At1g05610), trehalose-phosphatase synthase 2 (At1g16980), and pfkB-type carbohydrate kinase (At1g06730). However, in the *cfbp1* mutant, the triose phosphate supply is blocked, leading to low starch content in the chloroplast and to an increased sucrose/starch ratio [8]. Notably, *cyFBP* gene is induced in *cfbp1* mutant [8] as well as sucrose synthase (At5g20830). Starch biosynthesis and degradation pathways are positively regulated both in rosettes and in roots from *cyfbp*. We reported that *cyfbp* Arabidopsis mutant over-accumulates starch, without a reduction of soluble sugars in the cytosol due to *cyFBP* inactivation [8] and the transcriptomic analysis reveals that *APL4* gene (At2g21590), involved in starch synthesis is up-regulated in *cyfbp* background. The lack of cyFBP is leading to carbohydrate reallocation, this situation is including the *cFBP1* induction [8]. Curiously, important glycolysis enzymes as phosphofructokinases PFK2 (At5g47810) and PFK7 (At5g56630), glyceraldehyde-3-phosphate dehydrogenase (At1g79530 and At3g04120), phosphoglycerate kinase (At3g12780), and pyruvate kinase (At5g63680) were up-regulated in *cyfbp* roots. Based on all these results, we presume that the *cyFBP* disruption that provokes an over-accumulation of starch induces the expression of starch degradation and glycolysis-related genes. Arabidopsis mutants lacking the *PFK2* gene, accumulates less starch and more soluble sugars than wild-type plants [14], demonstrating the opposite biological function of *PFK2* and *cyFBP* during the regulation of sucrose synthesis, when PFK2 activity is stimulated cyFBP activity is inhibited. Recent studies have also demonstrated that a sucrose phosphate synthase double knockout Arabidopsis mutant (*spsa1*/*spsc*) has mechanisms to alleviate the blockage of the starch-to-S6P conversion process such as starch turnover acceleration, and channeling of starch breakdown products towards the glycolytic, oxidative pentose phosphate, and tricarboxylic acid cycle pathways [13].

The different expression of genes in rosette and roots, evidence a coordinated cross-talk between both organs in order to maintain a suitable plant development. In *cfbp1* and *cyfbp* mutants, 85% and 88% of genes are induced in roots respectively, whilst 44% and 68% are induced in rosettes. As in leaves, root transcriptome responds to the deficiencies provoked by the lack of the heterotrophic cyFBP or the photosynthetic cFBP1. The analysis of functional-gene categories revealed that *cFBP1* or *cyFBP* inactivation also induced expression changes of genes involved in RNA regulation, protein metabolism and modification, and biotic as well as abiotic stress responses. The *k*-means clustering analysis indicates that *cfbp1* and *cyfbp*-regulated genes are also induced in Arabidopsis plants subjected to biotic and abiotic stress (Additional file 7: Figure S3). Ma and Bohner [37], defined a common stress transcriptome that apparently represents universal cell-level stress responses by analyzing the stress-dependent expression profile in Arabidopsis. The plant responds to different types of stimuli by reprogramming the expression of similar metabolic pathways. The physiological and metabolic changes caused by *cFBP1* or *cyFBP* gene disruption probably lead the plants to a condition of permanent stress; this is corroborated by the number of antioxidant proteins identified in the proteomic analysis and the determination of the enzymes involved in the oxidative metabolism [8]. The situation of stress is clearly visible in *cfbp1*, given that the growth of this mutant appeared to be severely inhibited, as shown by Rojas et al. [8], whereas *cyfbp* plants displayed a wild-type phenotype.

In this study, we found that 63 genes related to proteasome were regulated. Proteolysis influences many metabolic activities such as biogenesis and maintenance of chloroplasts in plants [38]. It is well known that over 1,300 genes contribute to protein degradation via the ubiquitin-proteasome system (UPS) [39]. This process is responsible for removing or modifying most abnormal peptides and short-lived cell regulators. Therefore, the UPS affects many processes, including the cell cycle, signal transduction, transcription, and stress responses in plants [40].

The Arabidopsis genome encodes for more than 2,000 transcription factors [41]. In both mutants, we found a large number of transcription factors to be regulated, most of these belonging to the AP2/EREBP, bZip, bHLH, MYB, GATA, WRKY, C2C2(Zn) DOF, and C2H2 zinc finger transcription-factor families. Members of the bZip, bHLH, MYB, and DOF families have been reported to be major transcriptional regulators in light signalling [42], and involved in sugar signalling and carbon partitioning [43–47]. The AP2/EREBP family has been well-characterized [48] and diverse members of this family have functions in redox-regulation in the photosynthesis context, but also in the functional linkage between hormone-dependent signalling and sugar sensing [48].

Gene expression is a complex process, mRNA and protein abundance are affected by many cellular and physical processes, including transcription, post-transcriptional regulation, RNA degradation and splicing, translation, post-translational modification, and degradation of proteins. Changes in mRNA levels do not always lead to similar alterations in protein levels or enzyme activities. Comparative studies of mRNA and protein abundance performed so far indicate that the correlation across large data sets is typically modest [36, 49]. In our study, from a total number of 128 spots identified with differences at the protein level, 32 (25%) were found to be regulated simultaneously by microarray analysis. These proteins, such as rubisco activase (At2g39730), glutaredoxin-C9 (At1g28480), 26S proteasome subunit 4 (At2g20140), ATP-dependent Clp protease (At5g50920) involved in chlorophyll biosynthesis, and a vegetative storage protein (AT5G24780), are associated with important functional categories (Additional file 8: Table S5). The congruence between our transcriptome and proteome analysis is of about ~20%. The apparently low correlation between proteome and microarray data is consistent with the general observation that mRNA levels do not always correlate with protein levels because of post-transcriptional, translational, and post-translational regulations and protein turnover [49, 50]. In this sense, although *cFBP1* is induced in the *cyfbp* mutant, no differences at the protein level were observed [8], suggesting that translational regulation could be taking place. Studies comparing the overlap in differential expression patterns from both transcript and protein profiling in Arabidopsis have reported congruence lower to 5% [51], but also a congruence of 31.1% [52]. These results highlight the importance of integrating mRNA and protein data in order to gain more accurate knowledge of the complex regulation mechanisms triggered by plant cells in response to a loss of key genetic information. Additionally, low abundant proteins (e.g. transcription factors or signalling proteins) could escape from a general proteomic analysis if specific enrichment techniques are not used. Moreover, we have to take into account existing differences in the sensitivity between both techniques. In this sense, although cyFBP is induced at both transcript and protein levels in *cfbp1* [8] no protein induction has been found in the present work.

## Conclusions

This large-scale characterization of transcriptome and proteome profiles provides copious data relevant to a comprehensive general understanding of the molecular basis and regulatory mechanisms underlying carbon metabolism changes in *cfbp1* and *cyfbp* mutants. Our experimental data are sufficient to demonstrate that *cFBP1* and *cyFBP* are key genes that contribute to modulating the starch-sucrose content balance in the plant cell; and that *cfbp1* or *cyfbp* mutation affects other important pathways, especially the significant regulation of genes related to carbon metabolism, protein metabolism, RNA regulation, signalling, and stress responses. Additionally, we provide for the first time the proteomic characterization of these metabolic changes in *cfbp1* and *cyfbp* that occur in both photosynthetic and non-photosynthetic tissues and we attempt to design a comprehensive scenario of the effects on carbohydrate-dependent pathways. Further studies that characterize the specific modifications of these proteins will provide a unique insight into the metabolic pathways controlling the synthesis of both sucrose and starch, which are key compounds for agricultural products.

## Methods

### Plant growth

*Arabidopsis thaliana* plants (ecotype Col-0), *cfbp1* (Gabi-kat line 472G06)[53] and *cyfbp* (SALK line SALK_064456)[54] mutants in Col-0 background were grown in Murashige and Skoog (MS) medium and incubated at 22 °C under long-day conditions (16-h-light/8-h-dark) and with photosynthetically active radiation of 120 μmol photons m^–2^ s^-1^. The phenotypes of the mutants are caused by the disruption of the FBPase genes as it was proved by complementation experiments carried out in a previous study [8]. The mutant *cyfbp* is also referred as *fins1* in the work by Choo and Yoo [55]. Rosettes and roots from 12-day-old plants were separated, and immediately transferred to liquid nitrogen before storage at -80 °C.

### RNA extraction and microarrays

Arabidopsis DNA microarrays were provide by the Galbraith laboratory (University of Arizona, Tucson, AZ, USA) and were produced from a set of 70-mer oligonucleotides. This microarray contained 29,110 probes from the Operon Arabidopsis Genome Oligo Set Version 3.0 (Operon). This oligo set represents 26.173 protein-coding genes and 28.964 protein-coding gene transcripts and 87 miRNAs, and is based on the ATH1 release 5.0 of the TIGR Arabidopsis genome annotation database (http://www.tigr.org/tdb/e2k1/ath1/). Total RNA extraction from rosettes and roots of mutants and cDNA synthesis were conducted using the Bio-Rad and Clontech kits. DNA microarray hybridization was carried out as described previously [56].

At each sample, cDNA, obtained from both rosettes and roots of *cfbp1* and *cyfbp* mutants, was compared with wild-type plants. To maximize the statistical reliability of the data, three independent biological experiments were performed to compare the transcriptome of rosettes and roots of both *cfbp1* and *cyfbp* mutants (treatments) with their respective wild-type plants (control), the labelling of the two cDNA samples with either Cy5 or Cy3 fluorescent dye was reversed to prevent potential dye-related differences in labelling efficiency.

The slides were scanned using a GenePix 4100A chip reader/scanner (Axon Molecular Devices). Spot intensities from scanned slides were quantified, using the GenPix Pro 5.1 software (Axon). With this program, local background correction was conducted. GenPix Pro 5.1 output data files were used to perform the lowest intensity normalization, standard-deviation regularization, replicate analysis and dye-swap filtering, using the MIDAS computer program [57]. Normalization between different slides was carried out by centring [58]. Bootstrap analyses with SAM enabled us to identify the differentially expressed genes, using a cut-off of 1.5 and adjusting the delta value, FDR, and FSN to minimize the number of false-positive genes [59]. We conducted *k*-means clustering analysis to group the cDNA clones according to the similarity of their expression patterns, using MeV software available from TM4 Microarray Software Suite (http://www.tm4.org/) and the default options [57].

MapMan software version 3.5.0 was used to display expression profiles at the pathway level (http://mapman.gabipd.org/web/guest/mapman). For this, the averaged signals for treatments were expressed relative to those in wild-type plants of the same experiment, and the ratio averaged converted to a log_2_ scale. The overview figures were prepared using mapping file version Ath_AGI_TAIR9_Jan2010. Biological enrichment analyses were performed with AgriGO and GOrilla, both based on GO terms. Statistical analysis of functional categories (bins) was performed with the Over-Representation Analysis of PageMan based on two-tailed Fisher’s exact test [60].

### Microarray data validation by QRT-PCR

We selected 14 genes that had been differentially expressed in rosettes and/or roots of *cfbp1* and *cyfbp* mutants for confirmation by QRT-PCR (Table 2) as described in Soto-Suárez et al. [56]. The gene expression level was compared between microarray and QRT-PCR. Similarly, the microarray ratio for each gene analysed was normalised against the microarray ratio obtained for 18S ribosomal gene. This allowed direct comparison between the 18S ribosomal gene-normalized QRT-PCR ratio and the 18S ribosomal gene microarray ratio for each transcript investigated.

### Protein extraction, solubilization and IEF

Protein extraction from root and rosette tissue was performed by using TCA–acetone–phenol protocol [61]. The final pellet was suspended in protein solubilization buffer (9 M urea, 4% CHAPS, 0.5% TritonX100, and 100 mM DTT). Three biological replicates of the quantified protein were performed per sample [62]. Isoelectrofocusing (IEF) was carried out on Precast 17 cm IPG pH 5–8 linear gradient (Bio-Rad) strips. Proteins were separated in the pH range 4–7 using a three-step procedure: 15 min at 500 V, followed by 2 h at 10,000 V and a final step of 10,000 Vh to complete 60,000 Vh. After focusing, the strips were immediately run.

### 2-D electrophoresis, gel staining, image capture and analysis

Focused IPG strips (pH 5–8) were equilibrated by immersing them for 20 min first in 375 mM Tris–HCl, pH 8.8, containing 6 M urea, 2% SDS, 20% glycerol, and 2% DTT, and then in the same solution containing 2.5% iodoacetamide instead of DTT. The strips were then transferred onto vertical slab 12% SDS-polyacrylamide gels (Bio-Rad PROTEAN Plus Dodeca Cell) and electrophoresis run at 55 mA/gel until the dye front reached the bottom of the gel. After electrophoresis, the gels were silver-stained as described by Yan et al. [63] or with coomassie brilliant blue G-250 (Sigma). Gel images were captured, digitalized (Molecular Imager Pharos FX™ Plus multiImager System, Bio-Rad), and analyzed with PDQuest™ 2-D analysis software (Bio-Rad laboratories, Hercules, CA) using ten-fold over background as a minimum criterion for presence/absence. With this program, spot-intensity calibration, spot detection, background abstraction, matching, and the establishment of master-gel were performed. Protein staining of individual spots was quantified by calculation of spot volume after normalization of the image using the total spot volume normalization method multiplied by the total area of all the spots. Significant spots were manually excised and stored in milli-Q water at 4 °C until MALDI-TOF/TOF analysis.

Protein spots digestion and MALDI-TOF analysis were carried out in the UCO-SCAI proteomics facility, a member of Carlos III Networked Proteomics Platform, ProteoRed-ISCIII. The digestion protocols were performed as described previously [64]. For MALDI-TOF analysis, a combined PMF search (MS plus MSMS) was performed using GPS Explorer™ software v 3.5 (Applied Biosystems) over non-redundant NCBInr database using the MASCOT search engine (Matrix Science, London; http://www.matrixscience.com) following parameters reported previously [64].

## Additional files

Additional file 1: Table S1. List of *cfbp1* and *cyfbp* genes identified as differentially expressed in rosettes and roots by microarray analysis. (XLSX 549 kb)
Additional file 2: Table S2.A-F. Gene Clustering analyses. Six transcriptional patterns identified by k-means clustering analysis in *cfbp1* and *cyfbp* mutants*.* (ZIP 136 kb)
Additional file 3: Figure S1. PageMan display of coordinated changes of gene categories regulated *cfbp1* and *cyfbp* inactivation. (PDF 4098 kb)
Additional file 4: Table S3. List of *cfbp1* and *cyfbp* genes identified as differentially expressed in rosettes and roots by microarray analysis which are involved in the Calvin cycle, glycolysis, and starch metabolism. (CSV 9 kb)
Additional file 5: Table S4. Biological processes affected by *cfbp1* and *cyfbp* genes disruption following the Gene Ontology (GO) functional classification scheme. (XLSX 252 kb)
Additional file 6: Figure S2. MapMan representation of expression changes of *cfbp1* and *cyfbp* genes associated with biotic and abiotic stress responses. (PDF 2874 kb)
Additional file 7: Figure S3. Clusters of *cfbp1* and *cyfbp* regulated genes compared with data from microarray experiments used for studying stress responses. Upper panel shows the comparison with regulated genes from rosettes and lower panel shows the comparison with regulated genes from roots. (PDF 1677 kb)
Additional file 8: Table S5. List of identified protein spots in rosettes and roots of *cfbp1* and *cyfbp* mutants. (DOCX 59 kb)
Additional file 9: Figure S4. MapMan bin membership for spots representing protein levels changes in *cfbp1* and *cyfbp*. (PDF 635 kb)

## Abbreviations

cFBP1: chloroplastic fructose-1,6-bisphosphatase
cyFBP: cytosolic fructose-1,6-bisphosphatase
FBPase: fructose-1,6-bisphosphatase
TRX: thioredoxin
UPS: ubiquitin-proteasome system
