# Supplementary figures and images for: Transcriptomic and proteomic approach to identify differentially expressed genes and proteins in Arabidopsis thaliana mutants lacking chloroplastic 1 and cytosolic FBPases reveals several levels of metabolic regulation
Source: BMC Plant Biol. 2016 Dec 1;16:258. doi: 10.1186/s12870-016-0945-7 (PMC5134223; doi:10.1186/s12870-016-0945-7)

Figure S1

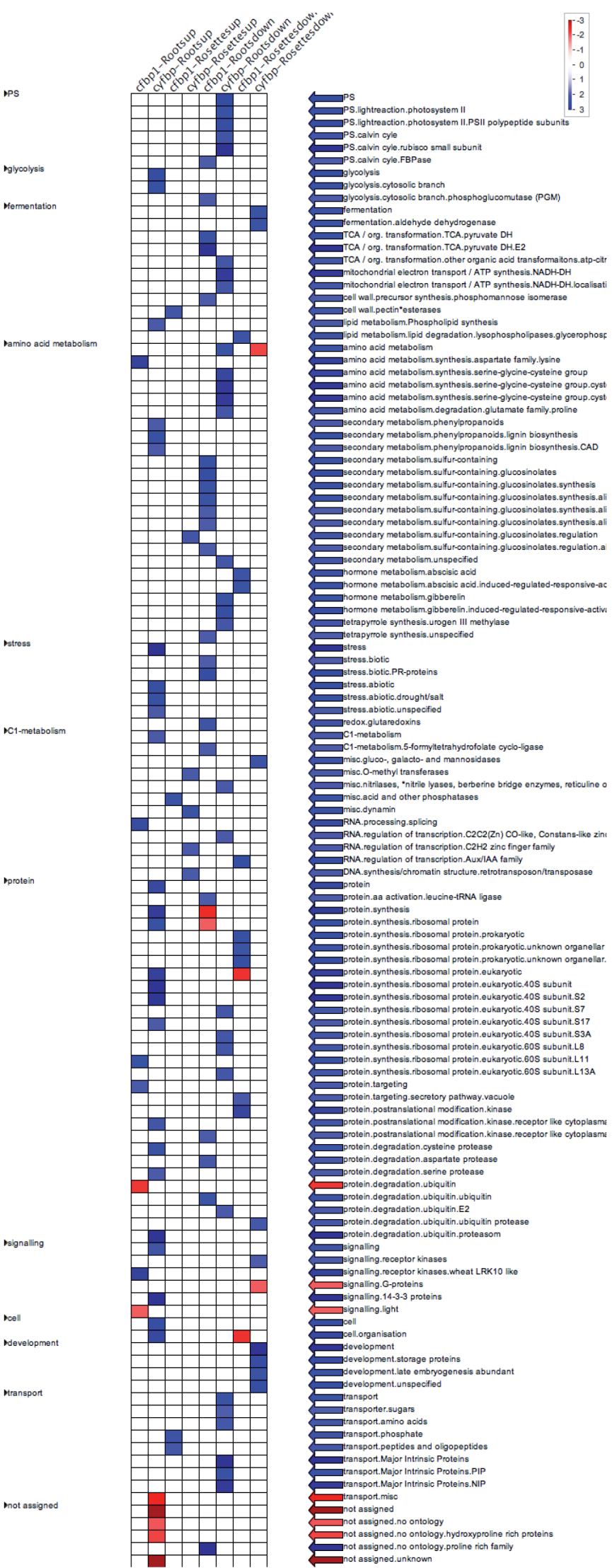

Supplement: Additional file 3: Figure S1. — PageMan display of coordinated changes of gene categories regulated cfbp1 and cyfbp inactivation. (PDF 4098 kb) [file 12870_2016_945_MOESM3_ESM.pdf]

Figure S2

***cfbfp1* - Rosettes**

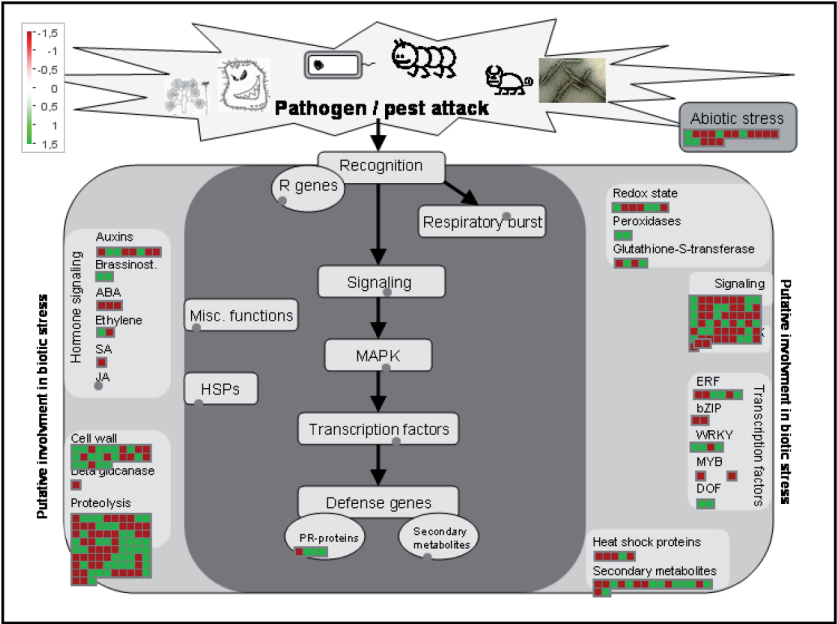

***cfbfp1* - Roots**

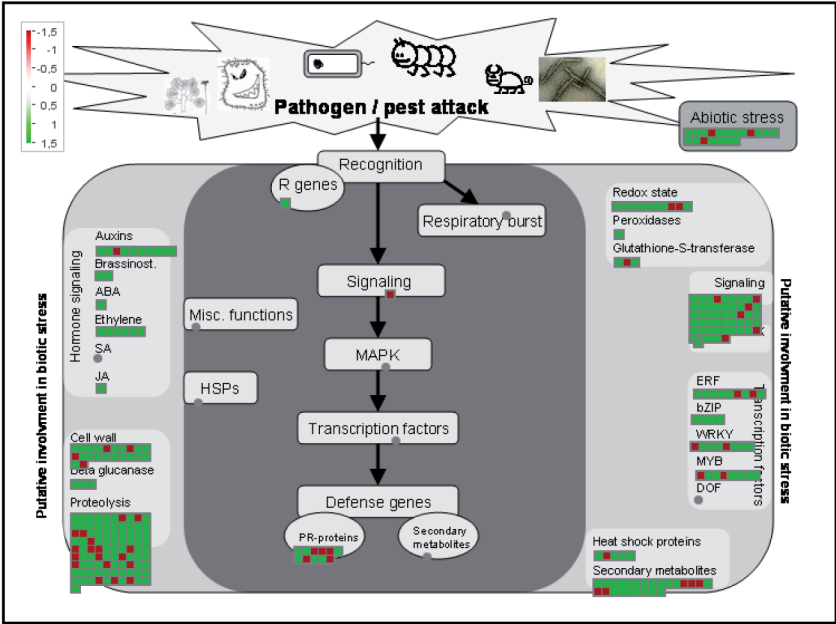

***cyfbp* - Rosettes**

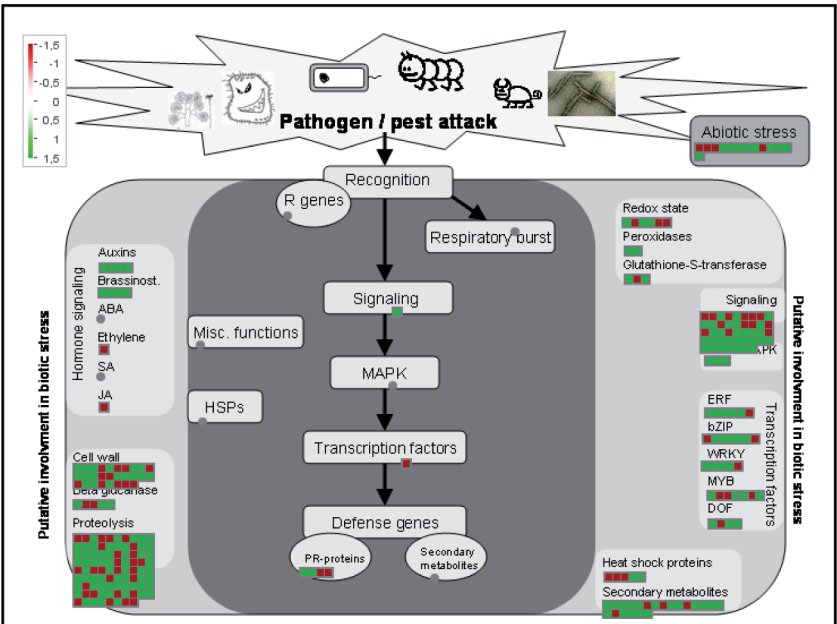

***cyfbp* - Roots**

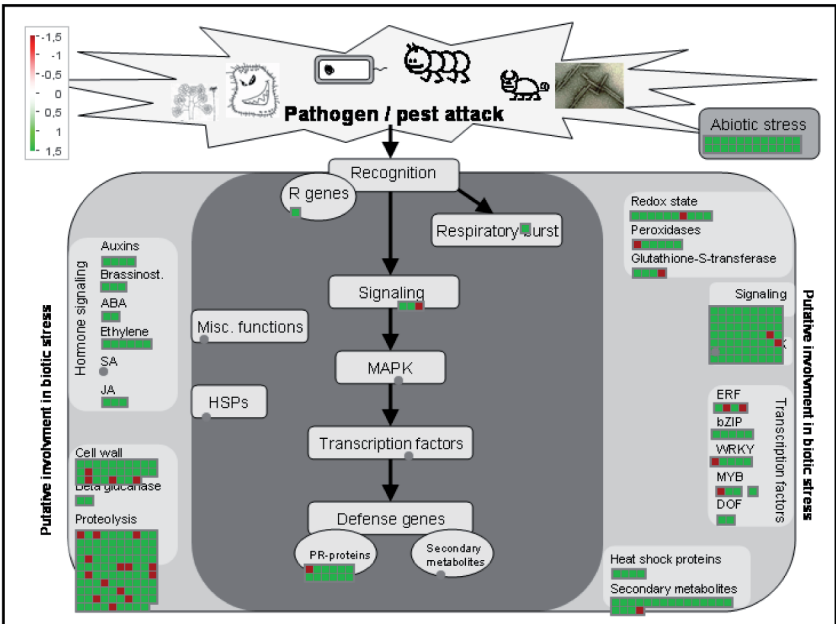

Supplement: Additional file 6: Figure S2. — MapMan representation of expression changes of cfbp1 and cyfbp genes associated with biotic and abiotic stress responses. (PDF 2874 kb) [file 12870_2016_945_MOESM6_ESM.pdf]

Figure S3

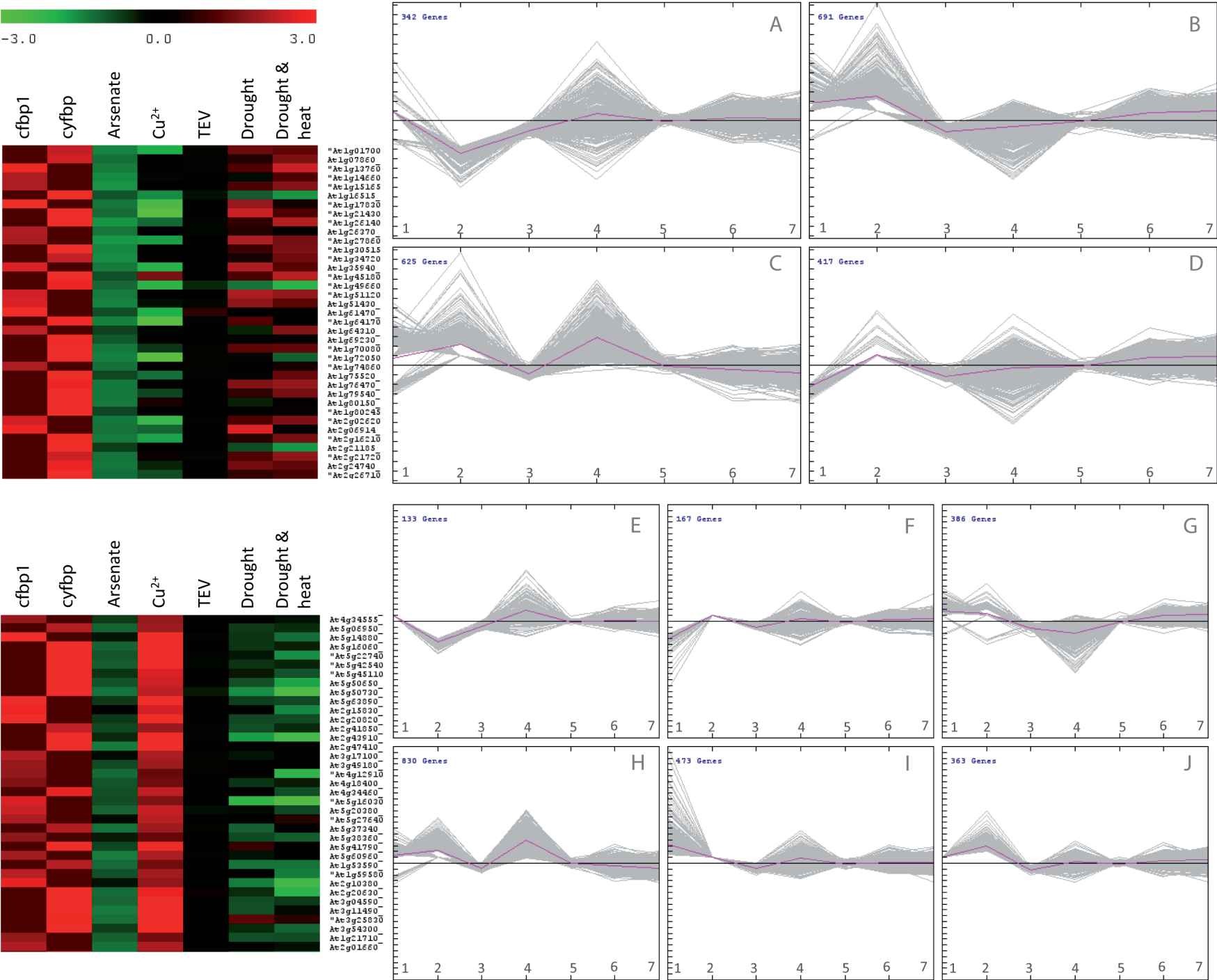

Supplement: Additional file 7: Figure S3. — Clusters of cfbp1 and cyfbp regulated genes compared with data from microarray experiments used for studying stress responses. Upper panel shows the comparison with regulated genes from rosettes and lower panel shows the comparison with regulated genes from roots. (PDF 1677 kb) [file 12870_2016_945_MOESM7_ESM.pdf]
