# Supplementary material for: Transcriptomic and proteomic approach to identify differentially expressed genes and proteins in Arabidopsis thaliana mutants lacking chloroplastic 1 and cytosolic FBPases reveals several levels of metabolic regulation
Source: BMC Plant Biol. 2016 Dec 1;16:258. doi: 10.1186/s12870-016-0945-7 (PMC5134223; doi:10.1186/s12870-016-0945-7)
Supplement: Additional file 8: Table S5. — List of identified protein spots in rosettes and roots of cfbp1 and cyfbp mutants. (DOCX 59 kb) [file 12870_2016_945_MOESM8_ESM.docx]

**Table S.5. List of identified protein spots in rosettes and roots of *cfbp1* and *cyfbp* mutants.**

^a^ Experimental and theoretical mass (*Mr*, kDa) and p*I* of identified proteins. Experimental values were calculated with PD-Quest software (BioRad). Theoretical values were retrieved from the protein database.

Spots with (^*^) indicate those that were found as differentially expressed with both microarray and proteomic analyses.

Spots with (^Ϯ^) indicate those that were predicted (with high confidence level) to be plastid-localized, according to Cell eFP Browser prediction for subcellular localization (<http://bar.utoronto.ca/cell_efp/cgi-bin/cell_efp.cgi>).

| **Spot** | **ID** | **AGI** | **Protein name** | **Sequence** | **Functional Categories GO** | **^a^Mr/pI experimental (theoretical)** | **No. of peptides** | **Mascot Scores** | |
| --- | --- | --- | --- | --- | --- | --- | --- | --- | --- |
|  |  |  |  |  |  |  |  | **Protein Score** | **Protein Score C.I. %** |
| ***cfbp1* mutant- Rosettes**  **Up-regulated spots** | | | | | | | | | |
| S1 ^Ϯ^ | CI-0803-S1 | ATCG00380 | Chloroplast Ribosomal protein S4 | LDNVIFRLGMAPTIPGAR | GO:0006412 translation | 74.0/5.2  (65.5/10.0) | 8 | 84 | 96.456 |
| S2^*^ | CI-1604-S2 | AT1G14860 | Nudix hydrolase 18 | CIPYRLKISSDGTISDEFEV | GO:0016787 hydrolase activity | 45.0/5.2  (48.0/5.3) | 11 | 171 | 99.711 |
| S3 | CI-4203-S3 | AT1G04400 | Cryptochrome 2 | VVFNHLYDPVSLVRDHNIK | GO:0003913 DNA photolyase activity | 33.0/7.2  (37.5/7.6) | 13 | 175 | 98.949 |
| S4^*^ | CI-6311-S4 | At1g28480 | glutaredoxin-C9 | MFGGLEKVMAAHISGEL  VPALK | GO:0045454 - cell redox homeostasis | 37.7/7.2  (38.5/6.8) | 6 | 149 | 95.199 |
| S5 | CI-0316-S5 | AT5G43600 | UAH ureidoglycolate amidohydrolase | VEFEGNGGHAGAVLMPARN | GO:0016811 hydrolase activity | 41.4/5.2  (45.3/6.0) | 15 | 179 | 99.902 |
| S6 | CI-0607-S6 | AT5G45020 | Glutathione S-transferase family protein | VNNESSEIIRMFNTEFNGIAKTP | GO:0000004; biological process unknown | 44.2/5.3  (33.8/4.7) | 8 | 172 | 99.038 |
| S7 | CI-1313-S7 | AT4G32160 | Phox (PX) domain-containing protein | RSKENLEQAIMSERERFNQMQWD | GO:0007165 signal transduction. GO:0035091 phosphatidylinositol binding | 38.6/5.4  (36.3/5.7) | 9 | 174 | 97.155 |
| S8 ^Ϯ^ | CI-1310-S8 | AT5G38520 | alpha/beta-hydrolase-like protein | MSRTATSTVNLRRISLRRDRVCV | GO:0008837 Amino-acid biosynthesis. regulation of Rab GTPase activity | 35.4/5.4  (41.1/7.6) | 5 | 184 | 98.182 |
| S9 ^Ϯ^ | CI-1305-S9 | AT3G53580 | Diaminopimelate Epimerase | VDNRDSSEPKITQEQAAKLCDRN | GO:0008837 Amino-acid biosynthesis. regulation of Rab GTPase activity | 38.4/5.6  (39.4/5.4) | 8 | 124 | 100 |
| S10 ^Ϯ^ | CI-1315-S10 | AT1G07570 | serine/threonine protein kinase | LLSGRRAVDKNRPSG | GO:0004674 protein serine/threonine kinase activity | 36.5/5.7  (32.2/7.9) | 8 | 178 | 95.124 |
| S11 | CI-1404-S11 | AT4G16835 | tetratricopeptide repeat domain-containing protein | ELAEFAAEKLLQLN | GO:0005488 binding | 40.8/5.1  (43.0/8.1) | 7 | 172 | 95.001 |
| S12^* Ϯ^ | CI-1503-S12 | AT3G52880 | monodehydroascorbate reductase (NADH)-like protein | FGVKGADSKNILYLREID | GO:0016656 oxidoreductase activity, acting on NADH or NADPH, quinone or similar compound as acceptor | 47.5/5.2  (48.3/5.3) | 23 | 696 | 100 |
| S13 | CI-1509-S13 | AT1G12050 | Putative fumarylacetoacetase | GSDSHFPIQNLPYGVFK | GO:0016823 hydrolase activity, acting on acid carbon-carbon bonds, in ketonic substances. | 44.9/5.3  (46.5/5.3) | 14 | 398 | 100 |
| S14^*^ | CI-2208-S14 | AT1G54270 | translation initiation factor 4A | RKGVAINFVTLDDQRMLF | GO:0006412 translation | 31.6/5.5  (26.5/8.0) | 18 | 170 | 99.675 |
| S15 | CI-2211-S15 | AT1G31812 | acyl-CoA binding protein | FGPVDTSRPGM | GO:0008289 lipid binding | 28.0/5.6  (24.9/5.9) | 19 | 161 | 97.236 |
| S16* ^Ϯ^ | CI-2405-S16 | AT2G39730 | Rubisco activase | ITRGKGMVDSVFQAPMGT | GO:0005524 ATP binding | 41.8/5.4  (52.3/5.8) | 19 | 669 | 100 |
| S17 | CI-2711-S17 | At4g18596 | pollen Ole e 1 allergen and extensin family protein | DSGCSDVSKEAYLRN | GO:0000004; biological process unknown | 62.0/5.4  (63.1/5.5) | 7 | 159 | 95.413 |
| S18 | CI-3119-S18 | At5g58750 | induced upon wounding stress-like protein | GRKLGVQVNETTMFDEGFW | GO:0006952 Defense response | 23.9/5.7  (20.3/4.6) | 7 | 152 | 96.895 |
| S19 | CI-3116-S19 | AT4G38680 | Cold shock protein 2 | MSGDNGGGERRKGSVKWF | GO:0003729 : mRNA binding | 23.9/5.6  (20.0/5.0) | 5 | 110 | 100 |
| S20 | CI-3214-S20 | At3g01290 | Hypersensitive-induced response protein 3 | EKAEAEKIIQIKRAEGEAESKY | GO:0006952 Defense response | 31.7/5.5  (31.5/5.6) | 12 | 245 | 100 |
| S21 | CI-6307-S21 | AT1G31650 | RHO guanyl-nucleotide exchange factor 14 | IMTPKARADIHM | GO:0000004; biological process unknown | 33.4/6.9  (31.8/10.6) | 10 | 152 | 95.859 |
| S22 | CI-7715-S22 | AT5G64440 | FAAH fatty acid amide hydrolase | VVIVGEDESPIDRLET | GO:0006952 Defense response | 58.9/7.2  (66.6/6.1) | 4 | 153 | 97.134 |
| S23 | CI-8508-S23 | AT5G63620 | GroES-like zinc-binding alcohol dehydrogenase family protein | YGAMAHAAEIRPGDSIAVIGIGGVG | GO:0004091carboxylesterase activity | 42.8/7.4  (44.3/7.5) | 6 | 140 | 100 |
| S24 | CI-3408-S24 | AT3G02230 | reversibly glycosylated polypeptide-1 | DDMWAGWCIKVICDHLGLGVKTGLP | GO:0016760: cellulose synthase (UDP-forming) activity | 41.0/5.7  (41.1/5.6) | 13 | 181 | 100 |
| S25 ^Ϯ^ | CI-3413-S25 | AT1G13440 | glyceraldehyde-3-phosphate dehydrogenase C2 (GAPC2) | AATYDEIKKAIKEESEGKMKGILGYT | GO:0006730 : one-carbon metabolic process | 41.9/5.7  (37.3/5.8) | 6 | 156 | 99.954 |
| **Down-regulated spots** | | | | | | | | | |
| S26 ^Ϯ^ | WT-0220-S26 | AT2G43090 | aconitase C-terminal domain-containing protein | TGKEYKLKPIGDAGPVIDAGGIFAYAR | GO:0008152 metabolic process | 26.4/5.2  (27.0/6.3) | 11 | 265 | 100 |
| S27 ^Ϯ^ | WT-7618-S27 | AT3G54660 | putative glutathione reductase | KYETEPSHDWTTLIANKNAELQRLT | GO:0006749 - glutathione metabolic process | 50.0/7.4  (54.2/6.3) | 19 | 326 | 100 |
| S28 ^Ϯ^ | WT-2114-S28 | AT1G76450 | Photosystem II reaction center PsbP family protein | NASEAFRVYTDETNKFEISI | GO:0000004; biological process unknown | 17.5/5.4  (16.6/5.5) | 10 | 230 | 100 |
| S29 ^Ϯ^ | WT-1116-S29 | At3g25770 | Allene Oxide Cyclase | SLQSISMTTLNNLSCNQQFHRSSLLG | GO:0008152 : metabolic process | 24.1/5.5  (21.2/5.6) | 7 | 194 | 100 |
| S30 ^Ϯ^ | WT-2316-S30 | AT5G35170 | adenylate kinase family protein | SSLSPSSTSLPLLQSPIRRRYRSLR | GO:0006163 : purine nucleotide metabolic process | 33.4/5.7  (39.4/8.6) | 9 | 153 | 97.730 |
| S31 ^Ϯ^ | WT-6202-S31 | AT4G25080 | magnesium-protoporphyrin IX methyltransferase | MPFAPSLLSSSSSVSQFLP | GO:0044237 cellular metabolic process | 31.1/6.8  (33.8/6.9) | 11 | 247 | 100 |
| S32 ^Ϯ^ | WT-6309-S32 | AT1G53240 | NAD-dependent malate dehydrogenase | IAAEIFKKAGMYDEKKLFGVTTLDVV | GO:0006730 : one-carbon metabolic process | 34.4/7.2  (36.0/8.5) | 7 | 229 | 100 |
| S33 ^Ϯ^ | WT-6422-S33 | AT5G67030 | zeaxanthin epoxidase precursor | GGLVFALAAKKKGFDVLVFEKDLSA | GO:0019761 glucosinolate biosynthetic process | 37.1/7.1  (40.6/5.5) | 4 | 145 | 98.268 |
| S34^*^ | WT-7106-S34 | At1g10630 | ADP-ribosylation factor | RILMVGLDAAGKTTILYKLKLGEIVTTIPTI | GO:0005525 : GTP binding | 18.5/7.5  (15.6/6.8) | 11 | 327 | 100 |
| S35^*^ | WT-8218-S35 | AT5G24780 | vegetative storage protein | HVQSSASVPGLIELLESNTIFGN | GO:0008134 transcription factor binding | 28.8/7.6  (30.4/5.3) | 6 | 196 | 99.999 |
| S36 | WT-8622-S36 | AT2G41930 | protein kinase domain-containing protein | APEIPETLPCDARLFLEKCFSRKP | GO:0004674 Protein serine/threonine kinase activity | 49.3/7.8  (50.6/6.5) | 10 | 166 | 99.009 |
| ***cfbp1* mutant- Roots**  **Up-regulated spots** | | | | | | | | | |
| S37 | CI-1705-S37 | AT2G48060 | hypothetical protein | LLVFILLWAVSTYIFNVAFSF | GO:0000004; biological process unknown | 66.0/5.3  (73.6/6.5) | 4 | 153 | 98.135 |
| S38^*^ | CI-3506-S38 | At2g28940 | protein kinase family protein | SNILLDERFGAKLSDFGLARQGPPEGLGH | GO:0005524 : ATP binding | 41.7/5.8  (48.5/5.0) | 11 | 163 | 98.043 |
| S39 ^Ϯ^ | CI-3522-S39 | AT1G63940 | Monodehydroascorbate reductase | IYDRMTRVEHVDHARRSAQHCVK | GO:0006950 : response to stress | 53.5/6.6  (54.3/6.6) | 24 | 467 | 100 |
| S40 ^Ϯ^ | CI-4503-S40 | At3g48730 | glutamate-1-semialdehyde 2,1-aminomutase 2 | RLARAFTGKQKFIKFEGCYHGHANSFL | GO:0006779 : porphyrin-containing compound biosynthetic process | 50.7/6.4  (50.5/6.4) | 9 | 125 | 100 |
| S41 ^Ϯ^ | CI-4602-S41 | AT2G07698 | F-type H+-transporting ATPase subunit alpha | MITRLFAQLVSLSIVT | GO:0046034 : ATP metabolic process | 55.2/5.9  (55.3/6.2) | 18 | 339 | 100 |
| S42 ^Ϯ^ | CI-4807-S42 | AT4g26970 | aconitase like protein | AAKGPLLLGVKAVIAKSFERIHRSNL | GO:0006101 : citrate metabolic process | 98.7/6.0  (99.4/5.8) | 24 | 445 | 100 |
| S43 | CI-6622-S43 | At1g78850 | putative glycoprotein (EP1) | TGDSSLVAYVKTPNANKK | GO:0045454 : cell redox homeostasis | 54.3/7.7  (49.8/7.2) | 7 | 169 | 100 |
| S44 ^Ϯ^ | CI-6803-S44 | At1g56070 | elongation factor EF-2 | MVKFTADELRRIMDYKHNIRNMSVIAH | GO:0003746 : translation elongation factor activity | 99.0/7.3  (95.0/5.8) | 5 | 87 | 99.993 |
| S45 | CI-6805-S45 | AT5G61780 | TUDOR-SN protein 2 | LLAAEARAIAGKKNIHSAKDS | GO:0009686 : gibberellin biosynthetic process | 100.0/7.2  (108.1/6.2) | 21 | 208 | 100 |
| S46 | CI-6815-S46 | At1g62720 | Pentatricopeptide repeat-containing protein | GLKPDVVSYTTMISGFCRKRQWDKSD | GO:0009845 : seed germination | 97.2/7.4  (101.0/8.7) | 8 | 157 | 95.855 |
| S47 | CI-7102-S47 | At1g19950 | HVA22-like protein h | SEIQRASSSKETIMEETLRITRGSLRK | GO:0000004; biological process unknown | 21.3/7.3  (17.0/8.2) | 5 | 161 | 96.826 |
| **Down-regulated spots** | | | | | | | | | |
| S48 | WT-1834-S48 | AT1G26810 | beta-1,3-galactosyltransferase 15 | DGCKDGYVVAHYQSPAEMTCLWRK | GO:0010493 : Lewis a epitope biosynthetic process | 94.0/5.2  (93.6/5.3) | 6 | 152 | 98.544 |
| S49 | WT-2028-S49 | AT3G61010 | ferritin subunit, putative | CLLCLPCPRCLHRERQCRLERFHQS | GO:0055114 : oxidation-reduction process | 28.2/5.7  (28.0/5.6) | 5 | 158 | 95.52 |
| S50 | WT-2717-S50 | AT3G50022 | hypothetical protein | KAYLRKHAPLTTDPGTMDEV | GO:0000004; biological process unknown | 59.6/5.4  (67.0/6.5) | 9 | 159 | 95.517 |
| S51^*^ | WT-2728-S51 | AT3G08590 | putative 2,3-bisphosphoglycerate-independent phosphoglycerate mutase | HSRLDQVQLLLKGFAERGAKRI | GO:0010118 : stomatal movemen | 60.9/5.5  (61.0/5.9) | 13 | 124 | 100 |
| S52^* Ϯ^ | WT-2823-S52 | AT5g50920 | ATP-dependent Clp protease | EIELQVTERFKERVVDEGY | GO:0010380 : regulation of chlorophyll biosynthetic proces | 93.3/5.3  (105.0/6.0) | 28 | 304 | 100 |
| S53 | WT-3025-S53 | AT3g10410 | carboxypeptidase Y-like protein | SELAVFYENGPFKITSNMSLAWN | GO:0006508 : proteolysis | 33.0/6.1  (30.6/5.8) | 6 | 155 | 98.149 |
| S54 ^Ϯ^ | WT-3716-S54 | At1g32900 | granule-bound starch synthase | QIKVGDKVENVRFFHCYKRGVDRV | GO:0009058 : biosynthetic process | 59.9/5.2  (60.8/5.8) | 5 | 163 | 98.088 |
| S55^*^ | WT-3719-S55 | At2g44160 | methylenetetrahydrofolate reductase MTHFR1 | SITYMAVNKGEQWVSNTA | GO:0055114 : oxidation-reduction process | 66.8/5.5  (60.1/5.3) | 7 | 108 | 100 |
| S56 | WT-3720-S56 | AT5g66760 | succinate dehydrogenase [ubiquinone] flavoprotein subunit 1 | HGFNTACITKLFPTRSHTVAAQGG | GO:0005524 : ATP binding | 70.2/5.8  (70.0/5.8) | 21 | 483 | 100 |
| S57 | WT-3817-S57 | AT1G23230 | hypothetical protein | MDQSQRTVAATPSSSRSY | GO:0000004; biological process unknown | 76.9/5.4  (56.1/5.7) | 8 | 170 | 99.636 |
| S58 ^Ϯ^ | WT-5424-S58 | AT5g54810 | tryptophan synthase beta chain | YRRENGEGPLIYLKREDLNHTGA | GO:0006979 : response to oxidative stress | 51.1/6.3  (51.8/6.2) | 7 | 104 | 100 |
| S59 ^Ϯ^ | WT-7117-S59 | At1g13280 | allene oxide cyclase | QHVPEKKGDRFEASYSFYFGDYGH | GO:0009695 : jasmonic acid biosynthetic process | 28.7/7.8  (27.9/9.1) | 6 | 181 | 99.972 |
| S60 | WT-7212-S60 | At5g02010 | RHO guanyl-nucleotide exchange factor 7 | HSSSVKRVLTAYVTKNEPRLKNLPLERS | GO:0005089 : Rho guanyl-nucleotide exchange factor activity | 41.9/7.7  (43.6/6.7) | 8 | 160 | 96.096 |
| S61 | WT-7519-S61 | AT5G35940 | jacalin lectin family protein | TVNYPYECITSVEGSYANT | GO:0000004; biological process unknown | 48.7/6.4  (50.1/6.4) | 15 | 310 | 100 |
| S62^* Ϯ^ | WT-8504-S62 | AT5G43780 | sulfate adenylyltransferase | RRVMKHEAETVPARIKLNRVDLEWVHV | GO:0008152 : metabolic process | 52.0/7.9  (52.1/8.8) | 8 | 141 | 95.324 |
| ***cyfbp*  mutant- Rosettes**  **Up-regulated spots** | | | | | | | | | |
| S63 | CY-3809-S63 | AT1G54620 | plant invertase/pectin methylesterase inhibitor | VSVHLQKAKEAPSICEMGFN | GO:0016023 : cytoplasmic membrane-bounded vesicle | 68.4/6.7  (70.1/6.8) | 6 | 140 | 95.023 |
| S64 ^Ϯ^ | CY-5029-S64 | AT3G56430 | uncharacterized protein | GLSEGMAKYGFLALS | GO:0000004; biological process unknown | 18.6/7.3  (22.0/7.0) | 5 | 147 | 95.867 |
| **Down-regulated spots** | | | | | | | | | |
| S65 | WT-4406 -S65 | At2g38760 | annexin D3 | HIASSLPFPLAKLLVTLAST | GO:0009651 : response to salt stress | 36.0/6.5  (36.2/6.6) | 8 | 151 | 95.386 |
| S66 ^Ϯ^ | WT-4410-S66 | At2g45350 | Pentatricopeptide repeat-containing protein | NMYASFGMWKDVRRVRTMMKE | GO:0009533 : chloroplast stromal thylakoid | 44.0/6.8  (46.2/7.1) | 9 | 155 | 95.995 |
| S67 ^Ϯ^ | WT-4710-S67 | AT5g24300 | soluble starch synthase | RDTVENFNPYAEGGAGTGTGWV | GO:0005975 carbohydrate metabolic process | 53.2/6.9  (56.4/7.5) | 16 | 308 | 100 |
| S68 | WT-5809-S68 | At4g37910 | heat shock protein 70 like protein | KVEAANKAVSKIGEHMSKGSGSSGS | GO:0009408 : response to heat | 63.0/7.0  (61.6/7.1) | 8 | 135 | 95.125 |
| S69 ^Ϯ^ | WT-6417-S69 | AT5g39830 | protease Do-like 8 | LTSLFMNLCFNPSRYLSALALGD | GO:0006508 : proteolysis | 44.9/7.1  (46.4/8.5) | 7 | 185 | 99.989 |
| S70^*^ | WT-7216-S70 | AT5G63400 | adenylate kinase 1 | AKKAVLTNIQAEKAPQEVTSEVKKALS | GO:0046939 : nucleotide phosphorylation | 27.0/7.3  (27.1/6.9) | 11 | 169 | 100 |
| S71 ^Ϯ^ | WT-7220-S71 | AT2G40800 | uncharacterized protein | DKVYRMTMRKINTAAEIL | GO:0000004; biological process unknown | 30.0/7.5  (30.2/8.6) | 11 | 130 | 96.677 |
| S72^*^ | WT-7306-S72 | AT1G17890 | GDP-L-fucose synthase | KPVYVILAAAKVGGIHANNTYPADF | GO:0008152 : metabolic process | 36.0/6.4  (36.3/6.2) | 7 | 113 | 100 |
| S73^*^ | WT-7308-S73 | AT3g25530 | glyoxylate reductase 1 | MNKSSYPPAFPLKHQQKDMRLALA | GO:0055114 : oxidation-reduction process | 35.1/7.3  (36.0/7.0) | 8 | 154 | 95.399 |
| S74 | WT-7624-S74 | AT1G53780 | 26S proteasome regulatory subunit T1 | ACFIRVVGSELVQKYIGEGARMV | GO:0006508 : proteolysis | 51.1/7.4  (48.6/7.1) | 10 | 151 | 95.722 |
| S75 | WT-7806-S75 | At2g15730 | nodulation protein-like protein | IQPWDIPYVHYPKPKTY | GO:0000004; biological process unknown | 67.0/7.2  (63.1/6.9) | 17 | 160 | 96.152 |
| S76 | WT-7822-S76 | AT2G23350 | poly(A) binding protein 4 | RKEERESAADKMKFTNVYVKNLSEATT | GO:0006413 : translational initiation | 72.0/6.3  (72.1/6.4) | 16 | 282 | 100 |
| S77 ^Ϯ^ | WT-8215-S77 | At3g27280 | prohibitin 4 | PNVAYLPGGQSMLFNLNPGR | GO:0006950 : response to stress | 31.0/6.6  (30.3/6.9) | 12 | 192 | 100 |
| S78 | WT-8811-S78 | AT4g37870 | phosphoenolpyruvate carboxykinase [ATP] | LESFGTPDFTIYNAGQFPCNRYTH | GO:0006094 : gluconeogenesis | 36.9/7.6  (32.6/7.8) | 11 | 180 | 99.961 |
| S79 | WT-8417-S79 | AT5G08300 | Succinyl-CoA ligase [GDP-forming] subunit alpha-1 | VVAFIAGLTAPPGRRMGHAGA | GO:0008152 metabolic process | 49.0/7.8  (41.9/8.5) | 10 | 442 | 100 |
| S80 | WT-8616-S80 | AT1G52100 | jacalin lectin family protein | TVNHPYEYITSVEGTYAHTQPYNCV | GO:0005529 sugar binding | 42.0/7.9  (42.0/8.1) | 27 | 597 | 100 |
| ***cyfbp*  mutant- Roots**  **Up-regulated spots** | | | | | | | | | |
| S81 ^Ϯ^ | CY-0211-S81 | AT4g20360 | chloroplast translation elongation factor EF-Tu precursor | SYTVTGVEMFQKILDEALAGDNVG | GO:0005525 : GTP binding | 26.4/5.3  (29.7/5.7) | 7 | 157 | 95.039 |
| S82^* Ϯ^ | CY-1102-S82 | AT1G42970 | glyceraldehyde-3-phosphate dehydrogenase B | AEAVGSGDPLEDFCKTNPADEECKVYD | GO:0006096 : glycolysi | 22.4/5.3  (20.1/5.1) | 10 | 149 | 95.844 |
| S83 ^Ϯ^ | CY-1104-S83 | At1g27450 | adenine phosphoribosyltransferase 1 | GVEARGFIFGPPIALAIGAKFVPMRKP | GO:0003999 : adenine phosphoribosyltransferase activity | 24.0/5.2  (19.5/5.5) | 10 | 284 | 100 |
| S84 | CY-1107-S84 | AT2G45790 | phosphomannomutase | ENGLVAHKDGKSIGIQSLKLHLGDDKL | GO:0019853 : L-ascorbic acid biosynthetic process | 25.2/5.3  (27.9/5.3) | 10 | 336 | 100 |
| S85 ^Ϯ^ | CY-1309-85 | AT3g44310 | nitrilase 1 | MSSTKDMSTVQNATPFNGVAPSTTVRV | GO:0000257 : nitrilase activity | 37.5/5.3  (37.9/5.4) | 7 | 137 | 100 |
| S86 ^Ϯ^ | CY-1406-S86 | At1g35720 | annexin-like protein | DHGEEILKSLEEGDDDDK | GO:0006979 : response to oxidative stress | 37.4/5.2  (37.3/4.7) | 4 | 157 | 95.209 |
| S87 | CY-1414-S87 | AT2G27020 | proteasome subunit alpha type-3 | VPDDLLEEAKTAAKTALEEMDAD | GO:0006508 : proteolysis | 27.9/5.1  (27.6/5.3) | 7 | 156 | 95.964 |
| S88 ^Ϯ^ | CY-1604-S88 | AT4g23100 | gamma-glutamylcysteine synthetase | ERFEWEKVMEGDKIIGLKQGKQ | GO:0006952 Defense response | 58.9/6.1  (59.0/5.9) | 14 | 249 | 100 |
| S89^*^ | CY-1608-S89 | AT1G72330 | alanine aminotransferase | LEEARSKGISVRALVVINPG | GO:0009058 : biosynthetic process | 52.2/5.3  (51.0/5.3) | 20 | 415 | 100 |
| S90^*^ | CY-1708-S90 | At1g54220 | dihydrolipoyllysine-residue acetyltransferase component 3 of pyruvate dehydrogenase complex | MAYASRIINHSKKLKDVSTLLR | GO:0008152 : metabolic process | 58.8/5.5  (57.1/5.3) | 15 | 354 | 100 |
| S91 | CY-1717-S91 | At3g03960 | TCP-1/cpn60 chaperonin family protein | EILEQLVETGSETMD | GO:0044267 : cellular protein metabolic process | 59.0/5.2  (59.4/5.2) | 18 | 384 | 100 |
| S92^*^ | CY-1902-S92 | AT3G09840 | cell division control protein 48-A | TVCIALADETCEEPKIRMNKVV | GO:0051301 : cell division | 91.4/5.3  (90.0/5.1) | 33 | 701 | 100 |
| S93^*^ | CY-2106-S93 | At1g17170 | glutathione S-transferase TAU 24 | IEYIDETWPDNNPLLPSDPY | GO:0004364 : glutathione transferase activity | 26.0/5.3  (25.1/5.6) | 11 | 244 | 100 |
| S94 | CY-2113-S94 | AT1G79210 | proteasome subunit alpha type-2-A | DKGPQLYQVDPSGSYFSWKA | GO:0000502 : proteasome complex | 25.0/5.4  (25.6/5.5) | 7 | 269 | 100 |
| S95 | CY-2201-S95 | At1g74900 | pentatricopeptide repeat-containing protein | KPDKAVKLFLNMHEHGCFQD | GO:0008380 : RNA splicing | 29.0/5.4  (30.2/5.6) | 9 | 154 | 95.854 |
| S96^*^ | CY-2202-S96 | AT2G41380 | S-adenosyl-L-methionine-dependent methyltransferase-like protein | AAKLPNVRYEITPPTMSSSEI | GO:0043414 macromolecule methylation | 30.0/5.3  (30.6/5.3) | 12 | 334 | 100 |
| S97^*^ | CY-2504-S97 | AT3G13920 | translational initiation factor 4A-1 | GVFSATMPPEALEITRKFMSK | GO:0003743 : translation initiation factor activity | 47.2/5.3  (46.9/5.4) | 17 | 396 | 100 |
| S98^*^ | CY-3101-S98 | At1g16920 | small GTP-binding protein (Rab11) | MAGYRVEDDYDYLFKVVLIGD | GO:0005525 : GTP binding | 24.4/5.4  (23.9/5.6) | 8 | 194 | 100 |
| S99^*^ | CY-3209-S99 | AT1G75280 | Isoflavone reductase-P3 | MATEKSKILVIGGTGYIGKF | GO:0006979 : response to oxidative stress | 33.2/5.7  (33.7/5.6) | 8 | 235 | 100 |
| S100 | CY-3401-S100 | AT1G74090 | desulfo-glucosinolate sulfotransferase | GCIHAQEFFQARPSDFLVCSYPK | GO:0019761 : glucosinolate biosynthetic process | 40.7/5.5 (40.7/5.5) | 18 | 424 | 100 |
| S101 ^Ϯ^ | CY-3511-S101 | AT5g41670 | 6-phosphogluconate dehydrogenase | QRPRSVIILVKAGAPVDQTIS | GO:0016491 : oxidoreductase activity | 52.4/5.5  (53.6/5.6) | 23 | 782 | 100 |
| S102 | CY-3609-S102 | At3g19130 | RNA-binding protein 47B | PDVTDVLLHETFSDRYPS | GO:0003723 : RNA binding | 49.4/5.6  (48.6/5.6) | 7 | 182 | 99.976 |
| S103^*^ | CY-3702-S103 | At2g44160 | methylenetetrahydrofolate reductase MTHFR2 | QNVVCVESMMHLTCTNMPVEK | GO:0055114 : oxidation-reduction process | 65.0/5.5  (66.8/5.5) | 24 | 379 | 100 |
| S104 | CY-4605-S104 | AT3g02090 | mitochondrial processing peptidase | ISAIGPIQDLPDYNKFRRRTYWNRY | GO:0006508 : proteolysis | 52.9/5.6  (53.7/5.7) | 17 | 575 | 100 |
| S105 ^Ϯ^ | CY-4709-S105 | At5g62530 | At5g62530 delta-1-pyrroline-5-carboxylate dehydrogenase 12A1 | LDPLNGEPFIKVAEVDESGTQPFVDS | GO:0072593 : reactive oxygen species metabolic process. | 59.4/6.0  (62.1/6.2) | 18 | 348 | 100 |
| S106^*^ | CY-5617-S106 | At2g20140 | 26S proteasome subunit 4 | DFKKAKEKVMFKKKEGVPEGLYM | GO:0000502 : proteasome complex | 49.0/5.8  (49.5/5.9) | 20 | 492 | 100 |
| S107^*^ | CY-6710-S107 | AT3G09260 | beta-glucosidase | MVLQKLPLIGLLLLLTIVASPA | GO:0006952 Defense response | 59.4/6.2  (60.1/6.2) | 21 | 529 | 100 |
| S108 | CY-6711-S108 | AT3G24170 | glutathione reductase, cytosolic | SSHGEEFVADVVLFATGRSP | GO:0006749 : glutathione metabolic process | 55.1/6.3  (54.2/6.3) | 14 | 278 | 100 |
| S109 ^Ϯ^ | CY-6901-S109 | AT4g33010 | glycine decarboxylase P-protein 1 (GLDP1) | DAVKPSDTFPRRHNSATPDE | GO:0006546 : glycine catabolic process | 74.4/6.0  (75.7/6.0) | 13 | 112 | 100 |
| S110^*^ | CY-6907-S110 | AT5G61780 | TUDOR-SN protein 2 | FQFVQVFVAGLQAPSMGRR | GO:0009686 : gibberellin biosynthetic process | 96.1/6.1  (97.2/6.3) | 30 | 465 | 100 |
| S111 | CY-7101-S111 | AT3g26340 | proteasome subunit beta type-5-A | VAADSRASMGGYISSQS | GO:0000502 : proteasome complex | 25.9/6.2  (29.7/6.0) | 11 | 288 | 100 |
| S112^*^ | CY-7203-S112 | AT1G17890 | GDP-L-fucose synthase | GSGSPLREFLHVDDLAD | GO:0008152 : metabolic process | 35.4/6.3  (35.5/6.6) | 11 | 428 | 100 |
| S113 ^Ϯ^ | CY-7302-S113 | AT1G75330 | ornithine carbamoyltransferase | DLKDFLAIDDFDTATIKTILDKA | GO:0006520 : cellular amino acid metabolic process | 37.5/6.3  (41.2/7.1) | 7 | 235 | 100 |
| S114 ^Ϯ^ | CY-7313-S114 | AT2G17265 | homoserine kinase | PLDLKPLRFPSDKDLFF | GO:0006952 Defense response | 38.5/6.4  (38.8/8.1) | 9 | 130 | 100 |
| **Down-regulated spots** | | | | | | | | | |
| S115 ^Ϯ^ | WT-0117-S115 | AT3G16640 | translationally controlled tumor protein-like protein | MLVYQDLLTGDELLSDSFPYKE | GO:0005515 : protein binding | 19.5/5.2  (18.9/4.5) | 9 | 223 | 100 |
| S116 | WT-2302-S116 | AT2G31390 | fructokinase | MLAGILRKNGVDDQGINFD | GO:0006014 : D-ribose metabolic process | 35.0/5.3  (35.4/5.3) | 16 | 344 | 100 |
| S117 | WT-3330-S117 | AT3G44320 | nitrilase 3 | SPQGQFLGKHRKVMPTSLERC | GO:0000257 : nitrilase activity | 34.0/5.3  (38.3/5.6) | 13 | 331 | 100 |
| S118 | WT-3313-S118 | AT4G33670 | D-threo-aldose 1-dehydrogenase | GLKALQVPRSDYIVATKCGR | GO:0019853 : L-ascorbic acid biosynthetic process | 35.5/5.6  (35.5/5.6) | 12 | 210 | 100 |
| S119 | WT-3914-S119 | AT1G18270 | ketose-bisphosphate aldolase class-II-like protein | DVYKPTLVRFENAGGLAANSPA | GO:0008152 : metabolic process | 97.0/5.6  (97.7/5.6) | 19 | 149 | 100 |
| S120^*^ | WT-7518-S120 | AT5g43940 | alcohol dehydrogenase class-3 | MATQGQVITCKAAVAYEPNKP | GO:0046292 : formaldehyde metabolic process | 40.5/6.6  (41.5/6.5) | 13 | 447 | 100 |
| S121 | WT-8210-S121 | AT4G26410 | hypothetical protein | ENSTSQFRSIQDFIPHALTQY | GO:0000004; biological process unknown | 30.0/7.0  (29.2/6.6) | 12 | 354 | 100 |
| S122 | WT-8314-122 | AT5G23250 | Succinyl-CoA ligase [GDP-forming] subunit alpha-2 | MSRQVTRLLGSLRHSGGGCS | GO:0008152 : metabolic process | 35.4/7.6  (36.5/8.5) | 7 | 161 | 100 |
| S123^* Ϯ^ | WT-8318-S123 | At5g61510 | NADPH2:quinone reductase | STVAVEESSEKKMVKG | GO:0016491 : oxidoreductase activity | 34.0/6.6  (34.9/6.4) | 12 | 205 | 100 |
| S124 | WT-8514-S124 | At5g06140 | sorting nexin 1 | MESTEQPRNISGSMQSPRSPS | GO:0035556 : intracellular signal transduction | 46.5/6.6  (46.6/6.7) | 20 | 262 | 100 |
| S125^*^ | WT-8514-S125 | At2g45240 | putative methionine aminopeptidase | IKTPEQIQRMRETCKIARE | GO:0016485 : protein processing | 45.4/7.2  (44.9/6.9) | 12 | 254 | 100 |
| S126 ^Ϯ^ | WT-8516-S126 | AT1G19920 | sulfate adenylyltransferase (ATP) | MSLMIRSSYVSHITLFQPRNSK | GO:0000103 : sulfate assimilation | 52.4/7.9  (52.1/8.0) | 14 | 256 | 100 |
| S127 | WT-8810-S127 | AT4g37870 | phosphoenolpyruvate carboxykinase [ATP] | NAAAAFAAVSEEERQKIQLQS | GO:0008152 : metabolic process | 73.0/6.6 (73.9/6.6) | 15 | 179 | 100 |
| S128 ^Ϯ^ | WT-8815-S128 | AT5g17920 | putative 5-ethyltetrahydropteroyltriglutamate--homocysteine S-methyltransferase | MASHIVGYPRMGPKRELKFALES | GO:0006950 : response to stress | 83.0/6.1 (84.6/6.0) | 10 | 189 | 99.992 |
