# Supplementary material for: Transcriptomic and proteomic approach to identify differentially expressed genes and proteins in Arabidopsis thaliana mutants lacking chloroplastic 1 and cytosolic FBPases reveals several levels of metabolic regulation
Source: BMC Plant Biol. 2016 Dec 1;16:258. doi: 10.1186/s12870-016-0945-7 (PMC5134223; doi:10.1186/s12870-016-0945-7)
Supplement: Additional file 9: Figure S4. — MapMan bin membership for spots representing protein levels changes in cfbp1 and cyfbp. (PDF 635 kb) [file 12870_2016_945_MOESM9_ESM.pdf]

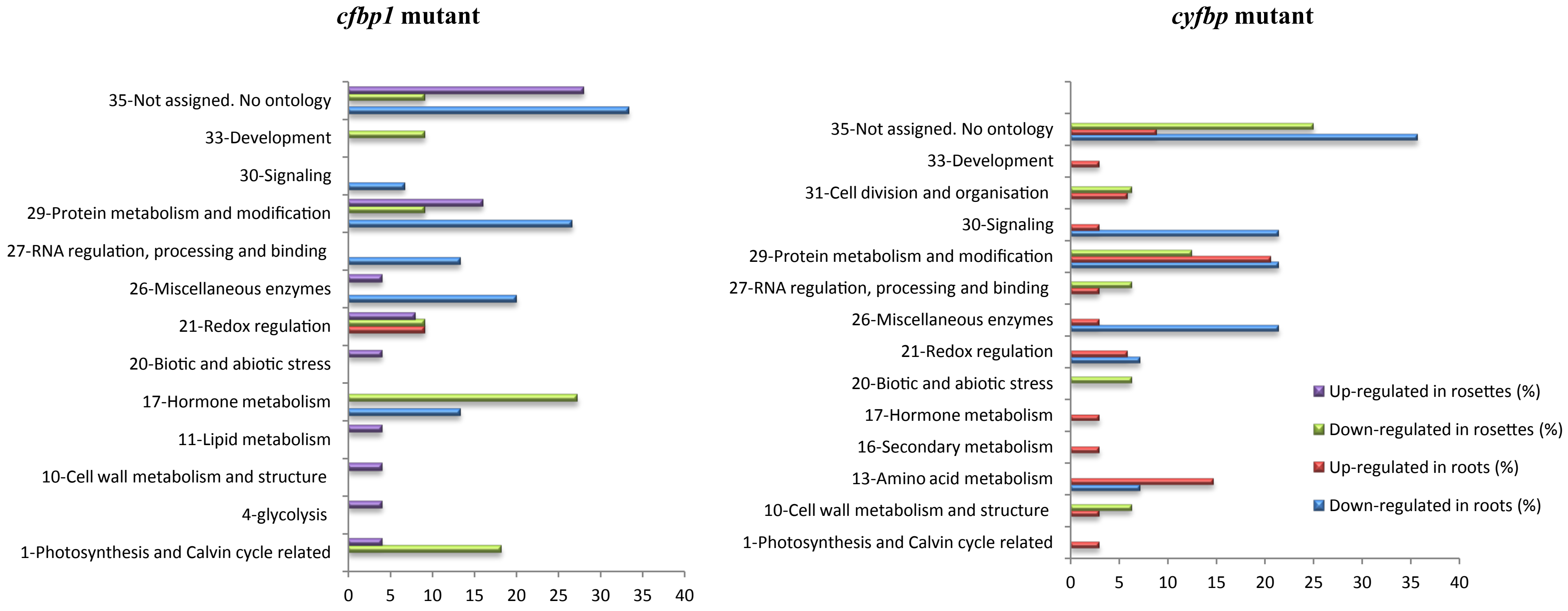

Figure S4. MapMan bin membership for differentially expressed protein spots in *cfbp1* and *cyfbp*.
The bin numbers and their corresponding bin name are graphed on the y-axis. Percentage of probe sets in each bin is graphed on the x-axis.
